# Nutrient-Dependent Endocycling in Steroidogenic Tissue Dictates Timing of Metamorphosis in *Drosophila melanogaster*

**DOI:** 10.1371/journal.pgen.1006583

**Published:** 2017-01-25

**Authors:** Yuya Ohhara, Satoru Kobayashi, Naoki Yamanaka

**Affiliations:** 1 Department of Entomology, Institute for Integrative Genome Biology, Center for Disease Vector Research, University of California, Riverside, Riverside, California, United States of America; 2 Life Science Center of Tsukuba Advanced Research Alliance, University of Tsukuba, Tsukuba, Ibaraki, Japan; Katholieke Universiteit Leuven, BELGIUM

## Abstract

Many animals have an intrinsic growth checkpoint during juvenile development, after which an irreversible decision is made to upregulate steroidogenesis, triggering the metamorphic juvenile-to-adult transition. However, a molecular process underlying such a critical developmental decision remains obscure. Here we show that nutrient-dependent endocycling in steroidogenic cells provides the machinery necessary for irreversible activation of metamorphosis in *Drosophila melanogaster*. Endocycle progression in cells of the prothoracic gland (PG) is tightly coupled with the growth checkpoint, and block of endocycle in PG cells causes larval developmental arrest due to reduction in biosynthesis of the steroid hormone ecdysone. Moreover, inhibition of the nutrient sensor target of rapamycin (TOR) in the PG during the checkpoint period causes endocycle inhibition and developmental arrest, which can be rescued by inducing additional rounds of endocycles by Cyclin E. We propose that a TOR-mediated cell cycle checkpoint in steroidogenic tissue provides a systemic growth checkpoint for reproductive maturation.

## Author Summary

Onset of sexual maturation constitutes a point of no return in animals; once this life-changing decision is made, upregulation of steroidogenesis leads to irreversible juvenile-to-adult transition in humans and insects alike. While nutrient signals contributing to this decision-making process have been well studied, molecular events that ultimately determine its precise timing remain a mystery. We report here that nutrient-dependent endoreplication, the replication of genomic DNA without cell division, in steroidogenic cells functions as an intrinsic timer, whereby degree of polyploidy sets the timing of reproductive maturation (i.e. metamorphosis) in fruit flies. The cumulative and irreversible nature of endoreplication thus provides an intrinsic molecular machinery underlying the irreversible decision-making process, which may be widely leveraged as a fundamental developmental timing mechanism.

## Introduction

Animals are heterotrophic and need to ingest nutrients from the environment during postembryonic development. Both availability and quality of food therefore are critical for timing animal growth and maturation. Since differentiation and functional maturation of each tissue are coordinated by the endocrine system, understanding how nutrient status affects hormonal states in developing animals is key to elucidating timing mechanisms responsible for juvenile-to-adult transition in animals.

In mammals, for example, activation of the hypothalamic-pituitary-gonadal axis triggers pubertal maturation [1]. Onset of puberty is controlled by multiple genetic and environmental factors, but the classical “critical weight hypothesis” points to the importance of body mass and nutritional state in activation of this neuroendocrine axis to initiate sexual maturation [2–4]. Similarly, in many holometabolous insects, the critical weight (CW) checkpoint needs to be surpassed before last instar larvae can initiate reproductive maturation, or metamorphosis, on a normal schedule [5]. In the fruit fly *Drosophila melanogaster*, CW is attained in the early half of the last (3rd) instar, after which starvation no longer delays the timing of metamorphosis [6, 7]. In fruit flies, CW virtually overlaps with another developmental checkpoint termed the minimal viable weight; larvae starved before this checkpoint do not initiate metamorphosis and eventually die [6, 7].

At the molecular level, attainment of CW is coupled with activation of steroidogenesis in *Drosophila*. After surpassing the CW checkpoint, production of the steroid hormone ecdysone is upregulated in a steroidogenic organ called the prothoracic gland (PG) [6–8]. Ecdysone, after conversion into its active form 20-hydroxyecdysone (20E) in peripheral tissues, then activates expression of downstream genes required for pupariation and subsequent metamorphic events [9]. Signaling pathways that couple nutritional status and steroidogenesis in the PG have been well investigated, and the importance of insulin signaling and target of rapamycin (TOR) signaling in the PG during the early third instar stage has been established in *Drosophila* [6–14]. However, considering CW as the “point of no return” in nutrition-dependent growth, CW attainment should require not only nutrient sensing machinery, but also a downstream molecular event leading to irreversible upregulation of ecdysone biosynthesis in the PG. The molecular nature of this invariable commitment of the PG cells to steroidogenesis has not yet been demonstrated.

Here we show that TOR-mediated progression of endocycle in the PG is required for activation of ecdysone biosynthesis that cannot be blocked by starvation in *Drosophila melanogaster*. Endocycle progression in the PG is strongly correlated with attainment of CW, and block of endocycle in the PG causes larval arrest due to reduction in ecdysone biosynthesis. Moreover, loss of TOR signaling in PG cells during the CW checkpoint period causes developmental defects due to endocycle arrest, which can be rescued by restoring Cyclin E expression in the PG. We propose that, in *Drosophila*, the evolutionarily-conserved, TOR-mediated cell cycle checkpoint in steroidogenic tissue can also function as a systemic growth checkpoint that triggers irreversible transition to metamorphosis.

## Results

### CW attainment is coupled with ecdysone biosynthesis

When reared under nutrient-rich conditions at 25°C (continuous feeding scheme), wild type *Oregon R* larvae undergo 1st-to-2nd instar molting, 2nd-to-3rd instar molting, and pupariation at around 48, 72, and 120 hours after egg laying (hAEL), respectively (S1A Fig). Under such conditions, more than 50% of larvae pupariate when starvation is initiated at around 78 hAEL or later, setting CW at about 0.7–0.8 mg (S1B, S1D and S1F Fig). If larvae are starved before this CW checkpoint from 72 hAEL, no pupariation is observed (S1D Fig). However, when larvae are starved from 72 to 120 hAEL and re-fed on nutrient-rich condition afterward (discontinuous feeding scheme), a modified CW of about 0.6 mg is attained at around 132 hAEL (S1C, S1E and S1G Fig). These two separate feeding schemes were used for subsequent analyses to observe the correlation of CW attainment and other physiological events.

**Fig 1:**
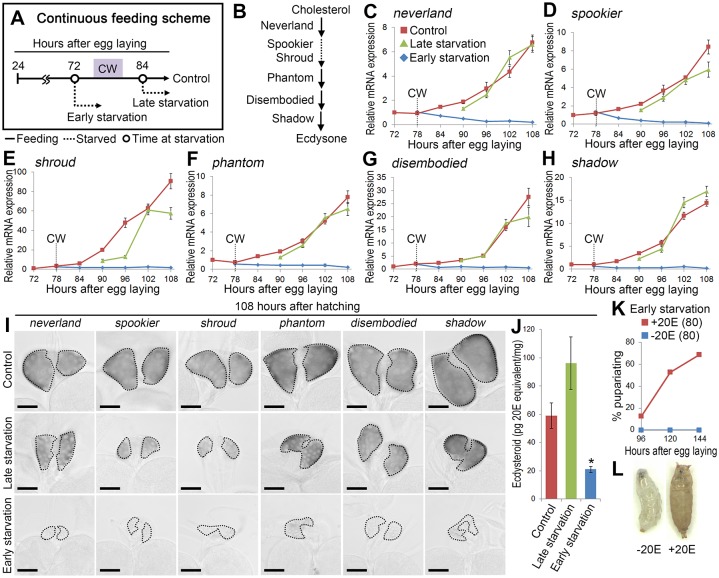
CW attainment is coupled with activation of ecdysone biosynthesis. **(A)** Schematic diagram of the continuous feeding scheme. Wild-type *Oregon R* larvae were reared on standard *Drosophila* medium (solid line) either continuously (control) or starved (dashed lines) from indicated time points. The CW checkpoint (78 hAEL) is indicated by a shaded box. **(B)** Schematic diagram of ecdysone biosynthetic pathway. **(C–H)** Starvation before CW attainment impairs increase in expression of ecdysone biosynthetic genes. Expression of ecdysone biosynthetic genes in control, late starved and early starved larvae was measured using qPCR. The CW checkpoint in control is indicated by dashed lines. Average values of five independent data sets are shown with standard errors. **(I)** Starvation before CW attainment causes decrease in expression of ecdysone biosynthetic genes in the PG. Whole-mount *in situ* hybridization was performed using antisense probes in control and starved larvae (late and early starvation) at 108 hAEL. The PGs are outlined by dashed lines. Scale bars, 50 μm. **(J)** Starvation before CW attainment causes decrease in ecdysteroid level. Whole-body ecdysteroid levels in control, late starved and early starved larvae at 108 hAEL were measured using ELISA. Average values of five independent data sets are shown with standard errors. Statistical significance was calculated using ANOVA with Tukey’s post hoc test (*P < 0.05). **(K)** 20E feeding rescues developmental arrest in early starved larvae. Larvae were starved on wet filter paper with or without 1 μg/ml 20E from 72 hAEL. Percentages of pupariated animals are shown at indicated stages. Numbers of animals tested are in parentheses. **(L)** Control starved larva (left) and pupariated animal by 20E feeding (right).

Using the continuous feeding scheme (Fig 1A), correlation of CW attainment and expression of ecdysone biosynthetic genes shown in Fig 1B [15] was investigated using quantitative RT-PCR (qPCR). Expression of the six ecdysone biosynthetic genes increased gradually after the CW checkpoint in larvae that were continuously fed (control) or starved after CW (late starvation; Fig 1C–1H). In contrast, their expression was not increased when larvae were starved before CW (early starvation), which was also confirmed using *in situ* hybridization (Fig 1I). Consistent with this, the ecdysteroid titer of early starved larvae was significantly lower than that of control and late starved larvae (Fig 1J). Moreover, developmental arrest caused by early starvation was significantly rescued when larvae were supplied with 20E-containing water (Fig 1K and 1L). These results indicate that, as suggested previously [9, 16], attainment of CW is tightly coupled with activation of ecdysone biosynthesis in the PG.

### CW attainment is correlated with endocycle activity in the PG

**Fig 2:**
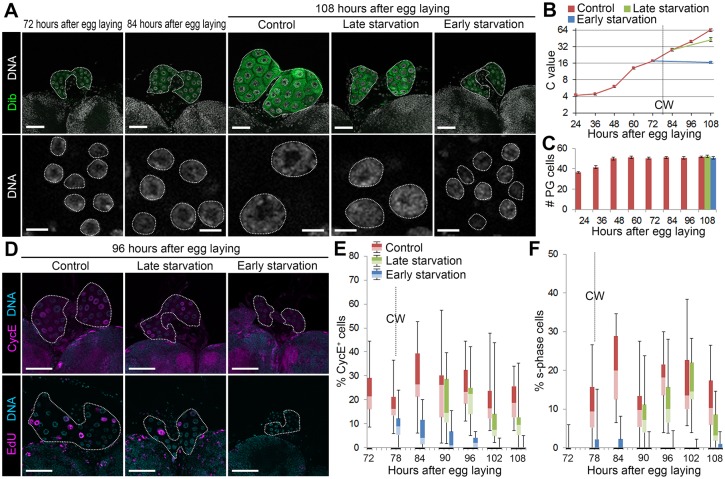
CW attainment is correlated with endocycle activity in the PG. **(A)** Starvation before CW attainment causes arrest of DNA content increase in the PG. The PGs (upper panels, outlined) and nuclei of PG cells (lower panels, outlined) were labeled for Dib (green) and DNA (white) at indicated stages of the continuous feeding scheme. Scale bars, 50 μm (upper panels) and 10 μm (lower panels). **(B and C)** The C value (B) and number (C) of PG cells of control, late starved and early starved larvae at indicated stages. The CW checkpoint in control is indicated by a dashed line in B. Average C value in control at 108 hAEL is normalized to 64C. Error bars represent standard errors. 10–17 PGs were analyzed for each group. **(D)** Starvation before CW attainment causes decrease in CycE expression and EdU incorporation in the PG. The PGs were labeled for DNA (blue) and CycE or EdU (magenta) at 96 hAEL. The PGs are outlined by dashed lines. Scale bars, 50 μm. **(E and F)** Percentages of CycE-positive (E) and EdU-positive (F) s-phase PG cells in control, late starved and early starved larvae at indicated stages. All data are shown as box plot, with a box representing lower and upper quartiles, a horizontal line representing median, and bars representing minimum and maximum data points. The CW checkpoint in control is indicated by dashed lines. 19–30 PGs were analyzed for each group.

A number of reports describe transcriptional regulators of ecdysone biosynthetic genes [17], but what ultimately ensures the irreversible decision by the PG to upregulate steroidogenesis after CW attainment? Since early starvation causes significant PG organ size decrease (Fig 1I), we speculated that the cell cycle system in the PG plays a critical role. PG cells undergo multiple rounds of endocycling, consisting of s-phase and gap phase [18, 19], leading to polyploid genomic DNA represented by chromatin values (C values) of greater than 4C. Consistent with previous studies [20, 21], we observed repeated rounds of endocycles in *Oregon R* PG cells during the 2nd and 3rd instars after mitotic cell cycles during the 1st instar (Fig 2A–2C). PG cells underwent two rounds of endocycles by the end of the 2nd instar, resulting in increase in the C value from 4C to 16C (Fig 2B). In the continuous feeding scheme, one round of endocycle progression was observed around the time of CW attainment in the early 3rd instar control larvae, and the C value of the PG cells increased to 64C through another round of endocycle (Fig 2A and 2B). Under the late starvation condition, the C value also increased close to 64C (Fig 2A and 2B). By contrast, the C value remained at 16C under the early starvation condition (Fig 2A and 2B). Importantly, very similar results were obtained in the discontinuous feeding scheme (S2A–S2D Fig).

We next observed expression of s-phase markers in the PG cells. Cyclin E (CycE), a nuclear protein triggering entry into s-phase [17, 18], was detected in nuclei of PG cells during the CW period and continued to be expressed thereafter under control and late starvation conditions (Fig 2D and 2E, S2E and S2F Fig). In contrast, expression of CycE was decreased after early starvation (Fig 2D and 2E, S2E and S2F Fig), suggesting that CycE-dependent s-phase entry is blocked in the PG if larvae are starved before the CW checkpoint. Using an s-phase marker 5-ethynyl-2’-deoxyuridine (EdU), we further confirmed that PG cells enter s-phase at the CW period, and s-phase PG cells were observed continuously thereafter in control and late starved animals (Fig 2D and 2F, S2E and S2G Fig). However, in the case of early starvation, PG cells failed to enter into s-phase (Fig 2D and 2F, S2E and S2G Fig). Taken together, these data show a strong positive correlation between endocycle activity in the PG cells and attainment of CW.

### Endocycle progression is required for ecdysone biosynthesis

**Fig 3:**
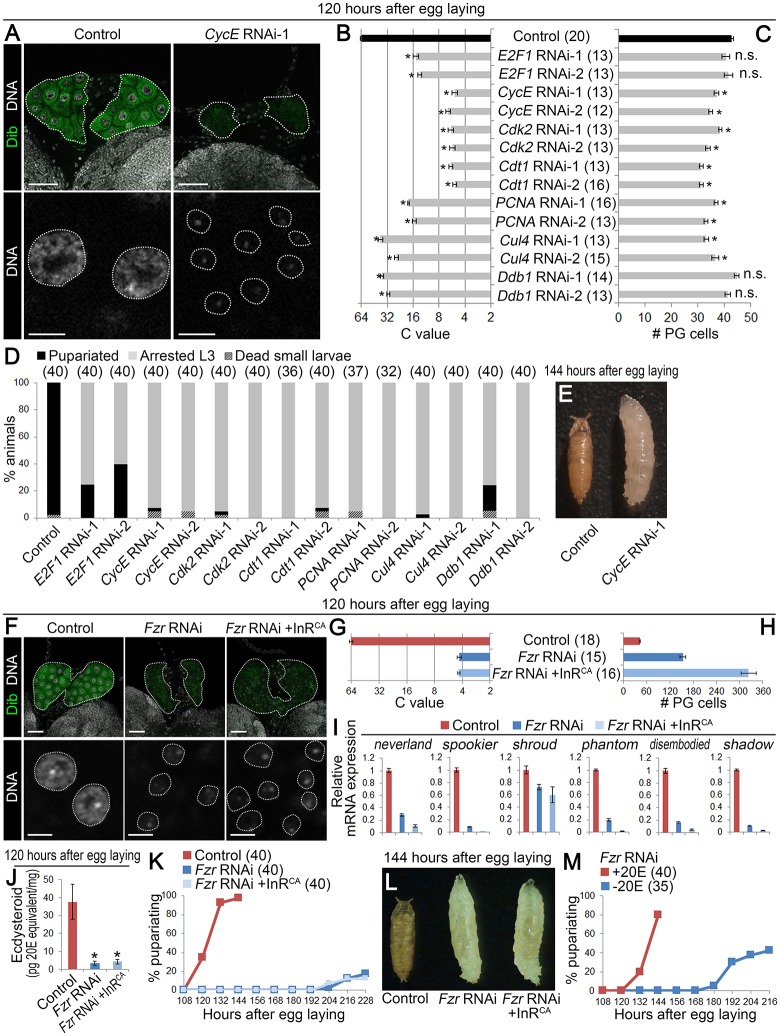
Endocycle is required for ecdysone biosynthesis. **(A)** Knockdown of *CycE* in the PG causes reduction in DNA content. The PGs (upper panels, outlined) and nuclei of PG cells (lower panels, outlined) of control (*phm22 > dicer2*) and *CycE* RNAi-1 (*phm22 > dicer2*, *CycE RNAi-1*) animals were labeled for Dib (green) and DNA (white) at 120 hAEL. Scale bars, 50 μm (upper panels) and 10 μm (lower panels). **(B and C)** Knockdown of endocycle regulators in the PG causes reduction in DNA content. Each gene was knocked down using two independent RNAi lines. The C value (B) and number (C) of PG cells of control (black bars) and RNAi (*phm22 > dicer2*, *RNAi*; gray bars) larvae at 120 hAEL. Average C value in control is normalized to 64C. Error bars represent standard errors. Numbers of animals tested are in parentheses. Significance was calculated using Student’s *t*-test (* *P* < 0.001; n.s., Not significant). **(D)** Knockdown of endocycle regulators in the PG causes arrest at the 3rd instar larval stage. Developmental profiles of control and RNAi animals are shown. Numbers of animals tested are in parentheses. **(E)** Pupariated control animal (left) and *CycE* RNAi-1 larva arrested at the 3rd instar stage (right). **(F)** Knockdown of *Fzr* in the PG causes block of mitotic-to-endocycle transition. The PGs (upper panels, outlined) and nuclei of PG cells (lower panels, outlined) in control (*phm22 > dicer2*), *Fzr* RNAi (*phm22 > dicer2*, *Fzr RNAi*) and *Fzr* RNAi +InR^CA^ (*phm22 > dicer2*, *Fzr RNAi*, *InR*.*A1325D*) animals were labeled for Dib (green) and DNA (white) at 120 hAEL. Scale bars, 50 μm (upper panels) and 10 μm (lower panels). **(G and H)** The C value (G) and number (H) of PG cells of control, *Fzr* RNAi and *Fzr* RNAi +InR^CA^ larvae at 120 hAEL. Average C value in control is normalized to 64C. Error bars represent standard errors. Numbers of animals tested are in parentheses. **(I)** Knockdown of *Fzr* causes reduction in expression of ecdysone biosynthetic genes. Expression of ecdysone biosynthetic genes in control, *Fzr* RNAi and *Fzr* RNAi +InR^CA^ larvae at 120 hAEL was measured using qPCR. Average values of three independent data sets are shown with standard errors. **(J)** Knockdown of *Fzr* causes decrease in ecdysteroid level. Whole-body ecdysteroid levels in control, *Fzr* RNAi, and *Fzr* RNAi +InR^CA^ larvae at 120 hAEL were measured using ELISA. Average values of five independent data sets are shown with standard errors. Statistical significance was calculated using Student’s *t*-test (* *P* < 0.05). **(K)** Knockdown of *Fzr* in the PG causes developmental arrest at the 3rd instar larval stage. Percentages of pupariated animals in control, *Fzr* RNAi and *Fzr* RNAi +InR^CA^ animals are shown at indicated stages. Numbers of animals tested are in parentheses. **(L)** Pupariated control animal (left) and *Fzr* RNAi and *Fzr* RNAi +InR^CA^ larvae arrested at the 3rd instar stage (middle and right, respectively). **(M)** 20E feeding rescues developmental arrest in *Fzr* RNAi. Percentages of pupariated *Fzr* RNAi animals reared on 20E-containing or control medium from 72 hAEL are shown at indicated stages. Numbers of animals tested are in parentheses.

Endocycle is regulated by multiple components as shown in S3A Fig, and inhibition of this oscillatory network blocks endocycle [18, 19, 22, 23]. To investigate the role of endocycling in the PG, the PG-selective *phm22-Gal4* was used to overexpress RNAi constructs and cDNA transgenes [24]. Compared to control (*phm22 > dicer2*), knockdown of *CycE*, *Cdk2*, and *Cdt1*, all essential components for s-phase initiation, resulted in severe reduction of the C value down to 4-8C at 120 hAEL (Fig 3A and 3B, S3B Fig). The C value of the PG cells was also decreased by knockdown of other regulatory components, including *E2F1*, *PCNA*, *Cul4*, and *Ddb1* (Fig 3B and S3B Fig), whereas the number of cells in the PG was only moderately affected by knockdown of some of the above endocycle components (Fig 3C). Endocycle inhibition in the PG also caused strong developmental arrest at the 3rd instar (Fig 3D and 3E), which was significantly rescued by 20E administration (S3C–S3P Fig). These data demonstrate that the inhibition of endocycling in the PG causes defective pupariation due to the lack of ecdysone.

Since cell size is usually coupled with ploidy, inhibition of endocycling in the PG cells resulted in reduction of not only the C value but also overall size of the PG (S3B Fig). This raises the possibility that developmental defects caused by endocycle inhibition can be explained simply by reduction of PG organ size. To test the importance of PG size for timing of pupariation, *fizzy-related* (*Fzr*), an essential gene for mitotic-to-endocycle transition [25], was knocked down in the PG. *Fzr* knockdown (*phm22 > dicer2*, *Fzr RNAi*) caused arrest of the C value at around 4C, which is equivalent to that in diploid cells before mitosis (Fig 3F and 3G). It instead caused a massive increase in cell number, which resulted in significant compensation of PG size (Fig 3F and 3H). In spite of this significant tissue size compensation, expression of ecdysone biosynthetic genes and ecdysteroid level were both significantly reduced in *Fzr* knockdown animals at 120 hAEL (Fig 3I and 3J). As a result, most of *Fzr* knockdown animals were still arrested at the 3rd instar, which could be rescued by 20E feeding (Fig 3K–3M). Moreover, overexpression of an active form of Insulin-like receptor (InR^CA^) in *Fzr* knockdown PG cells (*phm22 > dicer2*, *Fzr RNAi*, *InR*.*A1325D*) caused further increase in PG cell number to fully compensate the organ size reduction, but still failed to rescue the defective ecdysone biosynthesis and developmental arrest (Fig 3F–3L). These results clearly suggest that defects in ecdysone biosynthesis caused by endocycle inhibition in the PG cannot be explained by reduction in the PG size or cell number, and instead indicate that qualitative changes associated with endocycle progression in the PG lead to its functional maturation.

### TOR function is required for endocycle progression to activate ecdysone biosynthesis

It has been established that entry into s-phase is under the control of TOR signaling in various cell types [18, 26], and that TOR signaling regulates ecdysone biosynthesis in the PG of *Drosophila* [13, 14]. These reports prompted us to investigate the role of TOR as an upstream regulator of endocycling in the PG. We first examined the loss-of-function of TOR in the PG by overexpressing the toxic extended domain (TED) of TOR (TOR.TED), which acts in a dominant-negative fashion (hereafter referred to as TOR^DN^) [27]. Compared to Control-1 (*phm22 > +*, control for TOR^DN^), TOR^DN^ (*phm22 > TOR*.*TED*) animals stopped development at the 3rd instar, although they underwent molting to the 2nd and to the 3rd instar (S4A–S4D Fig). *TOR* RNAi in the PG caused indistinguishable phenotypes (S4C and S4D Fig). Consistent with this, expression of ecdysone biosynthetic genes was reduced in TOR^DN^ and *TOR* RNAi animals (S4E–S4J Fig), ecdysteroid level was decreased in TOR^DN^ animals (S4K Fig), and their pupariation was restored by 20E feeding from 72 hAEL (S4L–S4N Fig). These results are consistent with the previous report [13] and indicate that TOR activity is required for ecdysone biosynthesis.

**Fig 4:**
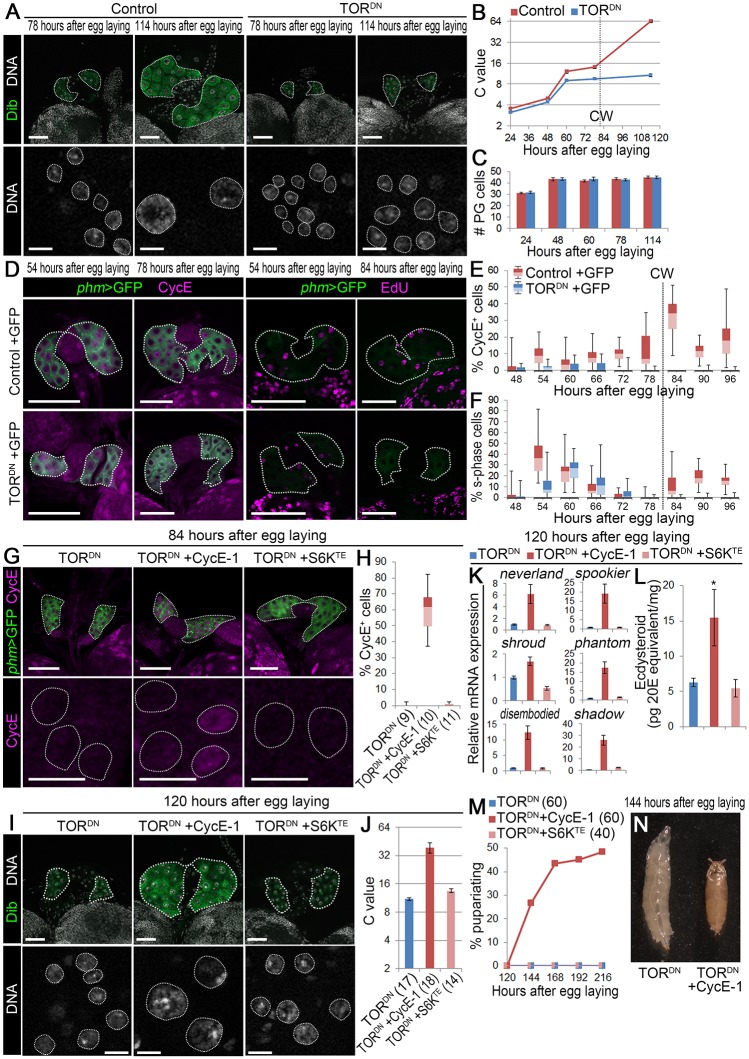
TOR is required for endocycle progression to activate ecdysone biosynthesis. **(A)** Expression of TOR^DN^ in the PG causes arrest in DNA content increase at the 3rd instar larval stage. The PGs (upper panels, outlined) and nuclei of PG cells (lower panels, outlined) of control (*phm22 > +*) and TOR^DN^ (*phm22 > TOR*.*TED*) animals were labeled for Dib (green) and DNA (white) at 78 and 114 hAEL. Scale bars, 50 μm (upper panels) and 10 μm (lower panels). **(B and C)** The C value (B) and number (C) of PG cells of control and TOR^DN^ larvae at indicated stages. The CW checkpoint in control (81 hAEL, see S4O Fig) is indicated by a dashed line in B. 11–19 PGs were analyzed for each group. Average C value in control at 114 hAEL is normalized to 64C. Error bars represent standard errors. **(D)** Expression of TOR^DN^ in the PG causes reduction in CycE expression and EdU incorporation at the 3rd instar larval stage. The PGs of control +GFP (*phm22 > mCD8*::*GFP*) and TOR^DN^ +GFP (*phm22 > mCD8*::*GFP*, *TOR*.*TED*) larvae were labeled for GFP (green) and CycE or EdU (magenta) at indicated stages. The PGs are outlined by dashed lines. Scale bars, 50 μm. **(E and F)** Percentages of CycE-positive (E) and EdU-positive (F) PG cells in control +GFP and TOR^DN^ +GFP larvae at indicated stages. All data are shown as box plot (see Fig 2E and 2F). The CW checkpoint in control is indicated by dashed lines. 17–26 PGs were analyzed for each group. **(G)** Expression of *CycE* in the PG of TOR^DN^ restores CycE protein expression. The PGs (upper panels, outlined) and nuclei of PG cells (lower panels, outlined) in TOR^DN^ +GFP, TOR^DN^ +CycE-1 +GFP (*phm22 > mCD8*::*GFP*, *TOR*.*TED*, *CycE-1*) and TOR^DN^ +S6K^TE^ +GFP (*phm22 > mCD8*::*GFP*, *TOR*.*TED*, *S6K*.*TE*) larvae were labeled for GFP (green) and CycE (magenta) at 84 hAEL. Scale bars, 50 μm. **(H)** Percentages of CycE-positive PG cells in TOR^DN^ +GFP, TOR^DN^ +CycE-1 +GFP and TOR^DN^ +S6K^TE^ +GFP larvae at 84 hAEL. All data are shown as box plot. Numbers of animals tested are in parentheses. **(I)** Expression of *CycE* in the PG of TOR^DN^ rescues reduction in DNA content. The PGs (upper panels, outlined) and nuclei of PG cells (lower panels, outlined) in TOR^DN^, TOR^DN^ +CycE-1 (*phm22 > TOR*.*TED*, *CycE-1*) and TOR^DN^ +S6K^TE^ (*phm22 > TOR*.*TED*, *S6K*.*TE*) animals were labeled for Dib (green) and DNA (white) at 120 hAEL. Scale bars, 50 μm (upper panels) and 10 μm (lower panels). **(J)** The C value of PG cells of TOR^DN^, TOR^DN^ +CycE-1 and TOR^DN^ +S6K^TE^ larvae at 120 hAEL. Average C value in TOR^DN^ is normalized to 11C, according to data in B. Error bars represent standard errors. Numbers of animals tested are in parentheses. **(K)** Expression of *CycE* in the PG of TOR^DN^ restores expression of ecdysone biosynthetic genes. Expression of ecdysone biosynthetic genes in TOR^DN^, TOR^DN^ +CycE-1 and TOR^DN^ +S6K^TE^ larvae at 120 hAEL was measured using qPCR. Average values of three independent data sets are shown with standard errors. **(L)** Expression of *CycE* in the PG of TOR^DN^ rescues decrease in ecdysteroid level. Whole-body ecdysteroid levels in TOR^DN^, TOR^DN^ +CycE-1 and TOR^DN^ +S6K^TE^ larvae at 120 hAEL were measured using ELISA. Average values of five independent data sets are shown with standard errors. Statistical significance was calculated using ANOVA with Tukey’s post hoc test (* *P* < 0.05). **(M)** Expression of *CycE* in the PG of TOR^DN^ rescues developmental arrest. Percentages of pupariated TOR^DN^, TOR^DN^ +CycE-1 and TOR^DN^ +S6K^TE^ animals are shown at indicated stages. Numbers of animals tested are in parentheses. **(N)** TOR^DN^ animal arrested at 3rd instar larval stage (left) and pupariated TOR^DN^ +CycE-1 animal (right).

We next examined whether TOR regulates endocycle progression. After mitotic cell cycles during the 1st instar (from 24 to 48 hAEL), the C value increased in the PGs of both control and TOR^DN^ animals by end of the 2nd instar (78 hAEL) (Fig 4A–4C). CycE and EdU-positive PG cells were also detected in both control and TOR^DN^ larvae during the 2nd instar (Fig 4D–4F). After molting to the 3rd instar, control larvae surpassed the CW of 0.7–0.8 mg at around 81 hAEL (S4O and S4P Fig), and the C value in the PG was increased from 16C to 64C during 78–114 hAEL (Fig 4A and 4B). In contrast, the C value in TOR^DN^-expressing PG did not increase after molting to the 3rd instar (Fig 4A and 4B). Likewise, *TOR* knockdown in the PG resulted in a decreased C value, but not the number of PG cells (S4Q–S4S Fig). Moreover, the percentage of CycE-positive and s-phase PG cells was decreased in the PG of TOR^DN^ animals during the CW checkpoint period, whereas the percentage of those cells started to increase at the CW period in control (Fig 4D–4F). In order to investigate the genetic interaction between *TOR* and *CycE* in the PG, we utilized *UAS-CycE-1* (type 1 CycE) construct, because of its moderate effect on cell cycle; although it has been known that continuous expression of *CycE* in endocycling cells blocks the progression of endocycle [28], *CycE-1* expression in the PG only led to modest increase in PG cell number and did not cause arrest of endocycle or pupariation (S4T–S4W Fig). In line with this, when *UAS-CycE-1* was introduced in TOR^DN^-expressing PG (*phm22 > TOR*.*TED*, *CycE-1*, referred to hereafter as TOR^DN^ +CycE-1), CycE protein was detected in 40–80%, but not all, of PG cell nuclei, indicating that CycE level is still oscillating (Fig 4G and 4H). As a result, the C value of PG cells, expression of ecdysteroid biosynthetic genes, and ecdysteroid level were significantly restored at 120 hAEL (Fig 4I–4L), and about 50% of TOR^DN^ +CycE-1 animals pupariated (Fig 4M and 4N). In clear contrast, although S6 kinase (S6K) is a well-known downstream target of TOR, overexpression of its active form (S6K^TE^) did not restore CycE expression or DNA content in TOR^DN^-expressing PG (Fig 4G–4J), and it did not rescue defects in ecdysone biosynthesis or developmental arrest of TOR^DN^ animals (Fig 4K–4M). Collectively, these results indicate that TOR signaling in the PG during the CW checkpoint period induces CycE expression in the PG to promote endocycling.

### TOR activates endocycle during CW period to initiate ecdysone biosynthesis

**Fig 5:**
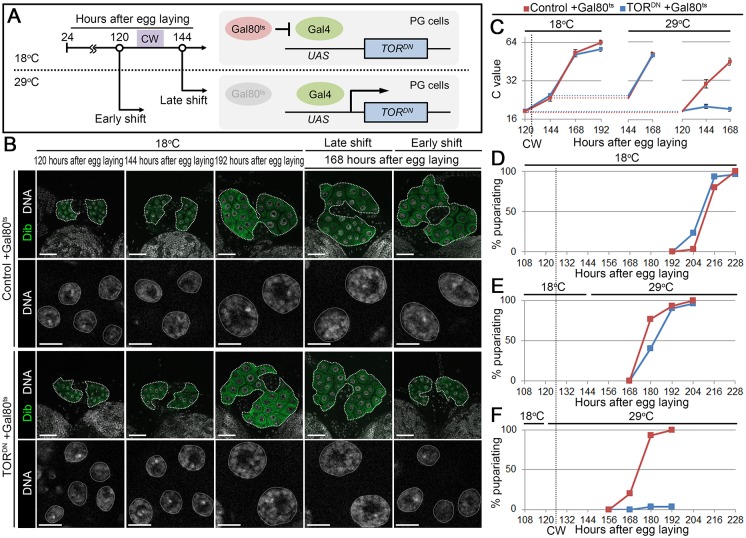
TOR is required for activation of endocycle in the PG at CW period. **(A)** Schematic diagram of the temperature-shift experiment. **(B)** Expression of TOR^DN^ before CW attainment causes arrest of DNA content increase. The PGs (upper panels, outlined) and nuclei of PG cells (lower panels, outlined) in control +Gal80^ts^ and TOR^DN^ +Gal80^ts^ animals were labeled for Dib (green) and DNA (white) at indicated stages. Scale bars, 50 μm (upper panels) and 10 μm (lower panels). **(C)** The C value of PG cells of control +Gal80^ts^ and TOR^DN^ +Gal80^ts^ larvae in the continuous 18°C (left), late shift (middle), and early shift (right) experiments at indicated stages. The CW checkpoint in control +Gal80^ts^ is indicated by a dashed line. Average C value of control +Gal80^ts^ at 192 hAEL in continuous 18°C experiment is normalized to 64C. Error bars represent standard errors. 12–18 PGs were analyzed for each group. **(D–F)** Expression of TOR^DN^ before CW attainment causes developmental arrest. Percentages of pupariated control +Gal80^ts^ and TOR^DN^ +Gal80^ts^ animals are shown at indicated stages in continuous 18°C (D), late shift (E), and early shift (F) experiments. The CW checkpoint in control +Gal80^ts^ is indicated by a dashed line. 30 animals were tested in each group.

To further confirm that TOR-mediated activation of endocycle is indeed required during the time of CW attainment, TOR^DN^ was expressed in the PG before or after CW attainment using the thermosensitive Gal80^ts^ system [29]. Gal80^ts^ protein, expressed under tubulin promoter (*tub-Gal80*^*ts*^), inhibits TOR^DN^ expression in the PG at 18°C, whereas Gal80^ts^ is inactivated and TOR^DN^ is expressed in the PG at 29°C (Fig 5A). At 18°C, control +Gal80^ts^ (*tub-Gal80*^*ts*^, *phm22 > +*) and TOR^DN^ +Gal80^ts^ (*tub-Gal80*^*ts*^, *phm22 > TOR*.*TED*) animals both attained CW at around 126 hAEL, when the body weight was about 0.8 mg (S5 Fig). Under this condition, the C value of PG cells increased to 64C in both control +Gal80^ts^ and TOR^DN^ +Gal80^ts^ animals (Fig 5B and 5C), and these animals pupariated successfully by 228 hAEL (Fig 5D). When animals were shifted to 29°C after the CW period (at 144 hAEL, indicated as late shift), the C value of PG cells increased equally in both control +Gal80^ts^ and TOR^DN^ +Gal80^ts^ larvae (Fig 5B and 5C), and both animals pupariated with comparable timing (Fig 5E). In contrast, a temperature shift before CW attainment (at 120 hAEL, indicated as early shift) caused arrest of the PG cell C value increase and development in TOR^DN^ +Gal80^ts^ (Fig 5B, 5C and 5F). These results are in excellent agreement with a previous report [13] and indicate that TOR function is required for endocycle activation at CW attainment to initiate invariable ecdysone biosynthesis.

### Insulin and Rag signaling pathways promote endocycling in the PG

**Fig 6:**
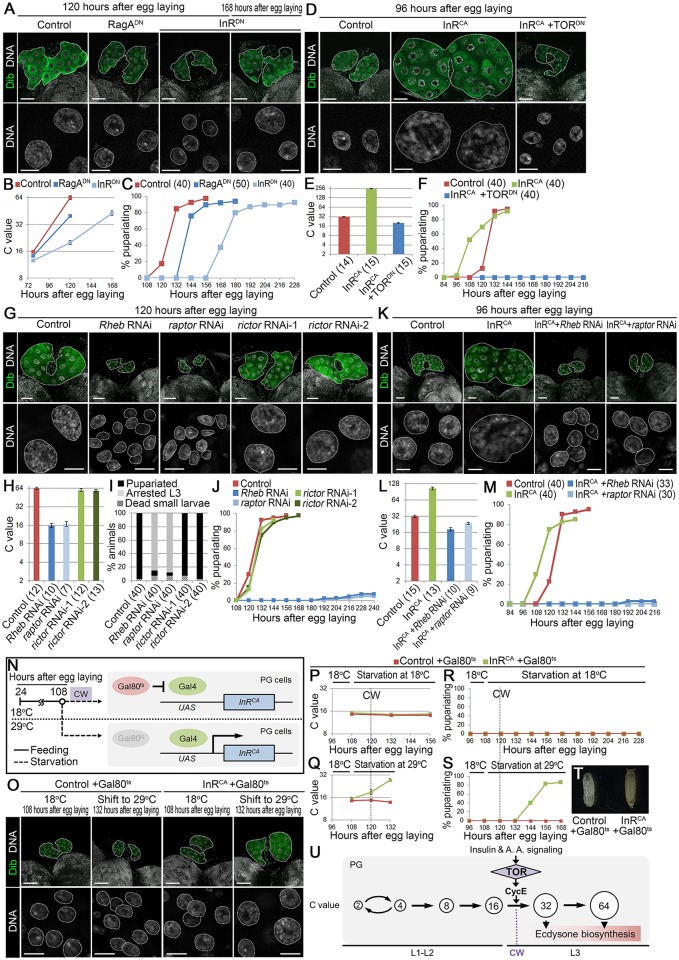
RagA and InR facilitate endocycling in the PG. (A) Expression of RagA^DN^ and InR^DN^ in the PG causes delay in DNA content increase. The PGs (upper panels, outlined) and nuclei of PG cells (lower panels, outlined) in control (*phm22 > +*), RagA^DN^ (*phm22 > RagA*.*T16N*) and InR^DN^ (*phm22 > InR*.*K1409A*) animals were labeled for Dib (green) and DNA (white) at indicated stages. Scale bars, 50 μm (upper panels) and 10 μm (lower panels). (B) The C value of PG cells of control, RagA^DN^ and InR^DN^ larvae at indicated stages. Average C value of control at 120 hAEL is normalized to 64C. Error bars represent standard errors. 11–16 PGs were analyzed for each group. (C) Expression of RagA^DN^ and InR^DN^ in the PG causes delay in pupariation. Percentages of pupariated control, RagA^DN^ and InR^DN^ animals are shown at indicated stages. Numbers of animals tested are in parentheses. (D) Expression of TOR^DN^ in the PG of InR^CA^ abolishes acceleration of DNA content increase. The PGs (upper panels, outlined) and nuclei of PG cells (lower panels, outlined) in control, InR^CA^ (*phm22 > InR*.*A1325D*) and InR^CA^ +TOR^DN^ (*phm22 > InR*.*A1325D*, *TOR*.*TED*) larvae were labeled for Dib (green) and DNA (white) at 96 hAEL. Scale bars, 50 μm (upper panels) and 10 μm (lower panels). (E) The C value of PG cells of control, InR^CA^ and InR^CA^ +TOR^DN^ larvae at 96 hAEL. Average C value in control at 96 hAEL is normalized to 32C, according to data in Fig 4B. Error bars represent standard errors. Numbers of animals tested are in parentheses. (F) Expression of TOR^DN^ in the PG of InR^CA^ abolishes acceleration of pupariation. Percentages of pupariated control, InR^CA^ and InR^CA^ +TOR^DN^ animals are shown at indicated stages. Numbers of animals tested are in parentheses. (G) Knockdown of *Rheb* and *raptor*, but not *rictor*, in the PG causes reduction in DNA content. The PGs (upper panels, outlined) and nuclei of PG cells (lower panels, outlined) in control (*phm22 > +*), *Rheb* RNAi (*phm22 > Rheb RNAi*), *raptor* RNAi (*phm22 > raptor RNAi*), *rictor* RNAi-1 (*phm22 > rictor RNAi-1*), and *rictor* RNAi-2 (*phm22 > rictor RNAi-2*) animals were labeled for Dib (green) and DNA (white) at 120 hAEL. Scale bars, 50 μm (upper panels) and 10 μm (lower panels). (H) The C value of PG cells of control, *Rheb* RNAi, *raptor* RNAi, *rictor* RNAi-1, and *rictor* RNAi-2 larvae at 120 hAEL. Average C value in control is normalized to 64C. Error bars represent standard errors. Numbers of animals tested are in parentheses. (I) Knockdown of *Rheb* and *raptor*, but not *rictor*, in the PG causes arrest at the 3rd instar larval stage. Developmental profiles of control, *Rheb* RNAi, *raptor* RNAi, *rictor* RNAi-1, and *rictor* RNAi-2 animals are shown. Numbers of animals tested are in parentheses. (J) Percentages of pupariated control, *Rheb* RNAi, *raptor* RNAi, *rictor* RNAi-1, and *rictor* RNAi-2 animals are shown at indicated stages. Numbers of animals tested are shown in E. (K) Knockdown of *Rheb* and *raptor* in the PG of InR^CA^ abolishes acceleration of DNA content increase. The PGs (upper panels, outlined) and nuclei of PG cells (lower panels, outlined) in control, InR^CA^ (*phm22 > InR*.*A1325D*), InR^CA^ +*Rheb* RNAi (*phm22 > InR*.*A1325D*, *Rheb RNAi*), and InR^CA^ +*raptor* RNAi (*phm22 > InR*.*A1325D*, *raptor RNAi*) larvae were labeled for Dib (green) and DNA (white) at 96 hAEL. Scale bars, 50 μm (upper panels) and 10 μm (lower panels). (L) The C value of PG cells of control, InR^CA^, InR^CA^ +*Rheb* RNAi, and InR^CA^ +*raptor* RNAi larvae at 96 hAEL. Average C value in control at 96 hAEL is normalized to 32C, according to data in Fig 4B. Error bars represent standard errors. Numbers of animals tested are in parentheses. (M) Knockdown of *Rheb* and *raptor* in the PG of InR^CA^ abolishes acceleration of pupariation. Percentages of pupariated control, InR^CA^, InR^CA^ +*Rheb* RNAi, and InR^CA^ +*raptor* RNAi animals are shown at indicated stages. Numbers of animals tested are in parentheses. (N) Schematic diagram of the temperature-shift experiment. (O) Expression of InR^CA^ triggers DNA content increase during starvation before the CW checkpoint. The PGs (upper panels, outlined) and nuclei of PG cells (lower panels, outlined) in control +Gal80^ts^ and InR^CA^ +Gal80^ts^ animals were labeled for Dib (green) and DNA (white) at indicated stages and temperature. Scale bars, 50 μm (upper panels) and 10 μm (lower panels). (P and Q) The C value of PG cells of control +Gal80^ts^ and InR^CA^ +Gal80^ts^ animals starved from 108 hAEL at 18°C (I) and 29°C (J). The CW checkpoint in control +Gal80^ts^ is indicated by a dashed line. The C value is normalized using that in control +Gal80^ts^ at 192 hAEL fed on standard *Drosophila* medium continuously at 18°C (see S6E and S6F Fig). Error bars represent standard errors. 13–18 PGs were analyzed for each group. (R and S) Expression of InR^CA^ in the PG of animals starved before the CW checkpoint triggers pupariation. Percentages of pupariated control +Gal80^ts^ and InR^CA^ +Gal80^ts^ animals starved from 108 hAEL at 18°C (K) and 29°C (L) are shown. The CW checkpoint in control +Gal80^ts^ is indicated by a dashed line. 30 animals were tested in each group. (T) Control +Gal80^ts^ animal arrested at the larval stage (left) and pupariated InR^CA^ +Gal80^ts^ animal starved from 108 hAEL at 29°C (right). (U) Model for the CW checkpoint mechanism in *Drosophila*. TOR-mediated endocycle progression in PG cells functions as an intrinsic timer that irreversibly activates ecdysone biosynthesis (see Discussion). A. A., amino acid.

TOR is under control of both insulin and RagA/C-mediated amino acid signaling, and insulin signaling in PG cells is particularly well-investigated as a nutrient sensing pathway coupled with CW attainment [6–8, 10–12, 16, 30]. To investigate whether these signaling pathways regulate endocycle progression in the PG, a dominant-negative form of InR (InR^DN^) or RagA (RagA^DN^) was expressed in the PG. The PG cell C value in control animals (*phm22 > +*) reached to 64C at 120 hAEL, whereas those in RagA^DN^ (*phm22 > RagA*.*T16N*) and InR^DN^ (*phm22 > InR*.*K1409A*) animals were around 32C and 16C at 120 hAEL, respectively (Fig 6A and 6B). In accordance with this, onset of pupariation was also delayed by 1 and 2 days in RagA^DN^ and InR^DN^ animals, respectively (Fig 6C). Moreover, their developmental delay was rescued by 20E administration (S6A and S6B Fig), suggesting that insulin and amino acid signaling pathways promote endocycle in the PG to activate ecdysone biosynthesis.

Although it is well-known that insulin acts through TOR signaling to regulate cell cycle and other cellular processes, some studies also indicate that insulin and TOR signaling pathways have retained distinct cellular functions in *Drosophila* [31–33]. Thus we examined whether insulin signaling activates endocycle through regulation of TOR in the PG. Both endocycle progression and timing of pupariation were accelerated in animals expressing InR^CA^ in the PG (*phm22 > InR*.*A1325D*) (Fig 6D–6F). When TOR^DN^ was introduced together in InR^CA^ animals (*phm22 > InR*.*A1325D*, *TOR*.*TED*), however, endocycle enhancement in the PG was abolished and animals were arrested at the 3rd instar (Fig 6D–6F). These data show that insulin signaling promotes endocycle progression through activation of TOR in the PG.

TOR forms two distinct protein complexes, TOR complex 1 (TORC1) and TOR complex 2 (TORC2) in *Drosophila* and other organisms [34]. TORC1 is regulated by both insulin and amino acid signaling pathways through their action on Ras homolog enriched in brain (Rheb), whereas TORC2 seems to be under the control of insulin signaling but not amino acid signaling [34, 35]. To investigate the function of TORC1 and TORC2 in the PG, *raptor* and *rictor* (TORC1- and TORC2-specific component, respectively), as well as *Rheb*, were knocked down in the PG. Knockdown of *Rheb* (*phm > Rheb RNAi*) and *raptor* (*phm > raptor RNAi*) in the PG caused significant reduction in the C value of PG cells, and *Rheb* and *raptor* RNAi animals were arrested at the 3rd instar larval stage (Fig 6G–6J). In clear contrast, knockdown of TORC2 component *rictor* (*phm > rictor RNAi-1* and *phm > rictor RNAi-2*) did not cause reduction in DNA content in the PG, and both *rictor* RNAi-1 and *rictor* RNAi-2 animals pupariated successfully on a normal schedule (Fig 6G–6J). These results suggest that TORC1 but not TORC2 is required for the progression of endocycle and ecdysone biosynthesis. Moreover, acceleration of endocycle and pupariation in InR^CA^ animals was abolished by knockdown of *Rheb* and *raptor* (Fig 6K–6M). Taken together, these results suggest that TORC1 has the major role in insulin signaling-mediated activation of endocycling and ecdysone biosynthesis in PG cells.

To further confirm that endocycling triggered by TORC1 signaling at the CW period initiates ecdysone biosynthesis in the PG, InR^CA^ was expressed in the PG of larvae starved before CW attainment using the Gal80^ts^ system (Fig 6N). Under the continuous feeding condition at 18°C, both control +Gal80^ts^ (*tub-Gal80*^*ts*^, *phm22 > +*) and InR^CA^ +Gal80^ts^ (*tub-Gal80*^*ts*^, *phm22 > InR*.*A1325D*) animals attained CW at around 120 hAEL when body weight was 0.6–0.7 mg (S6C and S6D Fig). Under this condition, no difference existed between C values of control +Gal80^ts^ and InR^CA^ +Gal80^ts^ PG cells at 192 hAEL (S6E and S6F Fig), and these animals pupariated by 216 hAEL (S6G Fig). When animals were starved before CW attainment (from 108 hAEL) at 18°C, the C value of PG cells did not increase in either control +Gal80^ts^ or InR^CA^ +Gal80^ts^ larvae (Fig 6P), and these animals were arrested at the larval stage (Fig 6R). In contrast, when animals were starved from 108 hAEL at 29°C, the C value of PG cells was close to 32C at 132 hAEL in InR^CA^ +Gal80^ts^, but not in control +Gal80^ts^ animals (Fig 6O and 6Q). InR^CA^ +Gal80^ts^ animals pupariated successfully by 168 hAEL (Fig 6S and 6T).

Taken together, our findings reveal the nutrient sensing mechanism and its associated molecular machinery that sets the timing of metamorphosis in *Drosophila*: the nutrient sensor TOR drives endocycling in the PG during the CW period, leading to irreversible activation of ecdysone biosynthesis to initiate metamorphosis (Fig 6U).

## Discussion

The endocycle is a ubiquitous cell cycle variant often coupled with cell growth and terminal cell differentiation, although its biological significance is diverse and not yet fully understood [18, 19]. In the present study, we demonstrated that endocycling of steroidogenic PG cells is required for functional maturation, leading to the high level of steroidogenesis critical for triggering metamorphosis in *Drosophila*. Interestingly, the high level of ecdysone biosynthesis necessary to induce metamorphosis is achieved only when PG cells engage in obligatory rounds (3–4 cycles) of endoreplication during the larval stage (Figs 2–4). Accordingly, when the rate of endocycle progression is suppressed in PG cells, timing of pupariation is proportionally delayed (Fig 6A–6C). The endocycle system in PG cells thus seems to function as an intrinsic timer, whereby degree of polyploidy sets the timing of the critical developmental transition (i.e. metamorphosis) in this holometabolous insect species. Considering the cumulative nature of endoreplication, it is reasonable to utilize endocycles in a postmitotic tissue as an internal measure of organismal growth and maturation. It would be interesting to investigate whether this unique function of endoreplication in *Drosophila* PG cells is more widely utilized as a critical developmental checkpoint among other multicellular organisms.

Our study also revealed that endocycling in PG cells is coupled with internal nutritional status through TOR signaling pathway during the CW checkpoint period (Figs 4–6 and S4–S6 Figs). The CW checkpoint is a built-in decision-making process, ensuring adequate nutrient uptake before metamorphosis in many holometabolous insect species [5, 36]. It is well known in *Drosophila* that nutrition sensing by the PG underlies this decision-making process [10–14], but how such decision is expressed at the cellular level has until now remained a mystery. Our model shown in Fig 6 proposes that the TOR-mediated cell cycle checkpoint couples insulin and amino acid signals with endocycles of PG cells, thereby translating nutritional status into stable expression of PG cell function. The irreversible nature of endoreplication thus provides a molecular and cellular basis for the irreversible CW checkpoint mechanism. It is noteworthy that *Drosophila* successfully converted an evolutionary conserved cell cycle checkpoint mechanism into a systemic, developmental checkpoint mechanism by utilizing endocycles in the steroidogenic tissue. Considering that steroid hormones control timing of systemic maturation in various metazoans, it is possible that a similar molecular mechanism operates in developmental checkpoints of other animal species.

How do endocycles lead to functional maturation of PG cells? Although our study indicates that organ size increase is not the major factor, the exact mechanism of how polyploidy is translated into the expression of cell function remains obscure. In many *Drosophila* endocycling cells, DNA replication in some euchromatic as well as heterochromatic regions can be incomplete, resulting in tissue-specific under-replication of these regions [37, 38], it is therefore possible that the PG-specific pattern of such biased DNA replication is generated by repeated rounds of endocycles, which in turn affects the PG-specific gene expression program. Detailed investigation of the replication protocol followed by PG cell genomic DNA is clearly warranted.

Although our results indicate that nutrition signals operate through TOR signaling pathway in PG cells, it is known that nutrition-dependent signaling components other than TOR, such as the forkhead box subclass O (FoxO) transcription factor working downstream of insulin signaling, also can regulate steroidogenesis in the *Drosophila* PG cells [16]. It is interesting to note, however, that FoxO also regulates endocycling in some cell types, such as the muscle [39]. These studies thus raise a possibility that the progression of endoreplication in PG cells is the core molecular event that governs overall timing of *Drosophila* metamorphosis. It will be important to examine the relationship between PG cell endoreplication and other molecular machineries and signaling pathways known to control expression of PG cell function [8, 14, 40, 41].

In summary, we have demonstrated the critical role steroidogenic cell endocycling plays as a timer for initiation of metamorphosis in *Drosophila*. In more general terms, our working model could offer unifying principles regarding how scheduling of critical developmental transitions is regulated, thereby providing a possible springboard for understanding operational mechanisms underlying systemic maturation processes in all animals.

## Materials and Methods

### *Drosophila* stocks

Detailed genotypes of the flies used in this study are summarized in S1–S3 Tables. To observe developmental profiles, flies were maintained in small cages and allowed to lay eggs for 24 hours on grape juice agar plates supplemented with yeast powder. Newly hatched larvae were transferred to Petri dishes with standard *Drosophila* medium. Larvae were cultured at 25°C under a 12-hour light/dark cycle, and developmental stages and lethality were scored periodically.

### CW measurement and starvation experiment

To examine CW, three independent groups of 10 larvae were collected at indicated time points. After measurement of the body weight of each group, larvae were transferred onto a piece of filter paper soaked in distilled water, and pupariated animals were scored at 12-hour intervals.

### qPCR

Total RNA was extracted from whole larvae using the QIAGEN RNeasy Mini Plus kit. Reverse-transcription was performed using SuperScript III (Invitrogen). cDNA was used as a template for qPCR using Quantifast SYBR Green PCR kit (QIAGEN) and Rotor-Gene Q (QIAGEN). The amount of target RNA was normalized using an endogenous control, *ribosomal protein 49* (*rp49*), and then the relative expression level was calculated (relative expression level = expression value of the gene of interest/expression value of *rp49*). Primer sets used for qPCR are shown in S4 Table.

### *In situ* hybridization

The same RNA probes against *neverland*, *spookier*, *shroud*, *phantom*, *disembodied*, and *shadow* transcripts prepared in the previous study [40] were used. Whole-mount *in situ* hybridization was performed as previously described [42].

### Ecdysteroid measurement

Fifteen larvae were rinsed with distilled water, and collected in a 1.5 ml microcentrifuge tube. The larvae were homogenized in 500 μl of methanol with a plastic pestle at room temperature. The samples were centrifuged at 15,000 g for 5 min at 4°C to obtain the supernatant. Ecdysteroid was quantitated by enzyme-linked immunosorbent assay (ELISA) using 20E EIA antiserum, 20E AchE tracer, and Ellman’s reagent (Cayman Chemical) as previously described [43].

### 20E feeding experiment

To rescue developmental arrest of *Oregon R* larvae during early starvation, larvae were transferred onto a piece of filter paper soaked in distilled water with 1 μg/ml 20E (Sigma) at 72 hAEL. Larvae starved on wet filter paper without 20E from the same time point were used as control. Pupariated animals were scored at 24-hour intervals.

In the rescue experiment of developmental arrest and delay, larvae were transferred to standard medium with 0.5 mg/g 20E at 72 hAEL. Larvae transferred to standard medium without 20E at the same time point were used as control. Developmental stages were scored at 12-hour intervals.

### Immunostaining

Larvae were dissected in phosphate buffered saline (PBS) and fixed for 25 min with 4% paraformaldehyde (PFA) in 0.1% PBT [0.1% Triton X-100 (Sigma) in PBS]. Tissues were washed with 0.1% PBT three times for 10 min each, washed with 1% PBT (1% Triton X-100 in PBS) for 5 min, blocked with 2% bovine serum albumin (Gemini bio-products) in 0.1% PBT for 30 min, and then incubated at 4°C overnight with primary antibodies diluted in blocking solution. Tissues were washed with 0.1% PBT three times for 10 min each, and incubated at 4°C overnight with secondary antibodies in 0.1% PBT. Together with the secondary antibody, Hoechst 33342 (Life technologies) was added at a 1:1500 dilution to detect DNA. After washing with 0.1% PBT three times for 10 min each, tissues were mounted in Vectashield mounting medium (Vector Laboratories).

The following primary antibodies were used at indicated dilutions: anti-Dib, 1:500; anti-CycE (Santa Cruz Biotechnology, sc33748), 1:500. Anti-Dib antibody was a gift from M. B. O’Connor [44]. Alexa Fluor 488- and Alexa Fluor 546-conjugated secondary antibodies were used to detect the primary antibodies.

### EdU incorporation experiment

EdU incorporation experiment was performed using Click-iT EdU 555 Imaging Kit (Life technologies). Larvae were dissected in Ringer’s solution, and tissues were incubated for 1 hour with 10 μM EdU in Ringer’s solution, fixed in 4% PFA in 0.1% PBT for 25 min. Fixed tissues were briefly washed twice in 0.3% PBT (0.3% Triton X-100 in PBS), washed in 0.3% PBT twice for 20 min each, blocked with 1% bovine serum albumin in 0.3% PBT for 30 min, and then incubated with Click-iT reaction cocktail including Alexa Fluor azide for 30 min. Tissues were washed briefly twice in 0.3% PBTw, washed in 0.3% PBT twice for 20 min each, and incubated at 4°C overnight with Hoechst 33342 (Life technologies) diluted at a 1:1500 in 0.1% PBT. In case of GFP staining, the primary antibody against GFP (Abcam, ab13970) was added at a 1:500 dilution, incubated 4°C overnight, washed with 0.1% PBT three times for 10 min each, and then incubated at 4°C overnight with Hoechst 33342 and the secondary antibody (Life technologies) in 0.1% PBT. After washing in 0.1% PBT three times for 10 min each, tissues were mounted in Vectashield (Vector laboratories).

### Image acquisition and analysis of fluorescent samples

Images were taken with a Zeiss Axio Imager M2 equipped with ApoTome.2. Image acquisition settings were as follows: 14 bits image depth, 1024 x 1024 pixels for CycE and incorporated EdU staining, and 2048 x 2048 pixels for DNA quantification. A series of 2D images was taken every 0.5 μm slices. Image analysis was performed using Fiji [45].

To distinguish PG cells stained with anti-CycE or incorporated EdU in control (*phm22 > +*) and TOR^DN^ (*phm22 > TOR*.*TED*) larvae, *UAS-mCD8*::*GFP* transgene was introduced both in control (*phm22 > mCD8*::*GFP*) and TOR^DN^ (*phm22 > mCD8*::*GFP*, *TOR*.*TED*) animals. PG cells of *Oregon R* were distinguished by their nuclei larger than surrounding cells in the ring gland. CycE-positive PG cells were defined as those in which nuclear CycE staining intensity was significantly stronger than cytoplasmic one. PG cells in s-phase were defined as those with pervasive EdU staining in the nucleus.

For DNA quantification, summation of DNA staining intensity in the PG was obtained from z-stacked images of the PG. PG cells were distinguished by Dib staining. DNA staining intensity in the PG was normalized using average DNA staining intensity in the brain lobe: DNA staining intensity in the PG/DNA staining intensity in the brain lobe. Normalized DNA staining intensity was divided by PG cell number to obtain DNA intensity per a PG cell. The C value of the control PG cells at 108–120 hAEL was set to 64C [21].

## Supporting Information

S1 Fig The CW checkpoint in wild-type *Oregon R* flies. **(A)** Developmental profile of *Oregon R* flies. Percentages of larvae and pupariated animals are shown at indicated stages. Mean percentages of three independent groups (10 animals in each group) are shown with standard errors. **(B and C)** Schematic diagrams of the starvation experiments. In the continuous feeding scheme (B), larvae reared on standard *Drosophila* medium (black line) were starved on wet filter paper (dashed lines) from indicated time points (white circles). In the discontinuous feeding scheme (C), larvae starved on wet filter paper from 72 to 120 hAEL were transferred to standard *Drosophila* medium, and re-starved from indicated time points (white circle). For each time point, three independent groups (10 larvae in a group) were weighed before starvation, and pupariated animals were counted during starvation. **(D**–**G)** The CW checkpoint in *Oregon R*. Percentages of pupariated animals after starvation at a given time point (D and E) and weight (F and G) are shown in the continuous feeding (D and F) and discontinuous feeding scheme (E and G). Mean percentages of three independent groups (10 larvae in each group) are shown with standard errors.
(TIF)

S2 Fig Endocycle activity of PG cells in discontinuous feeding scheme. **(A)** Schematic diagram of the discontinuous feeding scheme using wild-type *Oregon R* flies. **(B)** Starvation before CW attainment causes arrest of DNA content increase in the PG. The PGs (upper panels, outlined) and nuclei of PG cells (lower panels, outlined) were labeled for Dib (green) and DNA (white) at indicated stages of the discontinuous feeding scheme. Scale bars, 50 μm (upper panels) and 10 μm (lower panels). **(C and D)** The C value (C) and number (D) of PG cells at indicated stages. The CW checkpoint in control is indicated by dashed line in C. The C value is normalized against that of the PG in control at 108 hAEL (see Fig 2B). Error bars represent standard errors. 10–17 PGs were analyzed for each group. **(E)** Starvation before CW attainment causes a decrease in CycE expression and EdU incorporation in the PG. The PGs were labeled for DNA (blue) and CycE or EdU (magenta) at 156 hAEL. The PGs are outlined by dashed lines. Scale bars, 50 μm. **(F and G)** Percentages of CycE-positive (F) and EdU-positive (G) s-phase PG cells at indicated stages. The CW checkpoint in control is indicated by dashed lines. 16–30 PGs were analyzed for each group.
(TIF)

S3 Fig 20E feeding rescues developmental arrest caused by knockdown of Endocycle regulators in the PG. **(A)** Schematic diagram of interaction between endocycle regulators. **(B)** Knockdown of endocycle regulators in the PG causes reduction in DNA content. Each gene was knocked down using two independent RNAi lines. The PGs (upper panels, outlined) and nuclei of PG cells (lower panels, outlined) of control (*phm22 > dicer2*) and RNAi (*phm22 > dicer2*, *RNAi*) animals were labeled for Dib (green) and DNA (white) at 120 hAEL. Scale bars, 50 μm (upper panels) and 10 μm (lower panels). **(C-P)** *E2F1* RNAi-1 (C) and 2 (D), *CycE* RNAi-1 (E) and 2 (F), *Cdk2* RNAi-1 (G) and 2 (H), *Cdt1* RNAi-1 (I) and 2 (J), *PCNA* RNAi-1 (K) and 2 (L), *Cul4* RNAi-1 (M) and 2 (N), and *Ddb1* RNAi-1 (O) and 2 (P) animals were reared on 20E-containing (0.5 mg/g 20E) or control medium from 72 hAEL. Percentages of pupariated animals are shown at indicated stages. Numbers of animals tested are in parentheses.
(TIF)

S4 Fig Detailed analyses of TOR pathway in ecdysone biosynthesis and endocycle progression in the PG. **(A and B)** Expression of TOR^DN^ in the PG causes developmental arrest at the 3rd instar larval stage. Percentages of larvae and pupariated animals in control-1 (*phm22 > +*) (A) and TOR^DN^ (*phm22 > TOR*.*TED*) (B) are shown at indicated stages. Mean percentages of three independent groups (10 larvae in each group) are shown with standard errors. **(C)** Knockdown of *TOR* in the PG causes developmental arrest at the 3rd instar larval stage. Developmental profiles of control-1, TOR^DN^, control-2 (*phm22 > dicer2*), *TOR* RNAi-1 (*phm22 > dicer2*, *TOR RNAi-1*), and *TOR* RNAi-2 (*phm22 > dicer2*, *TOR RNAi-2*) animals are shown. Numbers of animals tested are in parentheses. **(D)** Pupariated control-1 and control-2 animals as compared to TOR^DN^, *TOR* RNAi-1, and *TOR* RNAi-2 animals arrested at the 3rd instar larval stage. **(E–J and E’–J’)** Inhibition of *TOR* in the PG causes reduction in expression of ecdysone biosynthetic genes. Expression profiles of ecdysone biosynthetic genes were measured using qPCR. Average values of triplicate data sets are shown with standard errors. **(K)** Expression of TOR^DN^ in the PG causes decrease in ecdysteroid level. Whole-body ecdysteroid levels in control and TOR^DN^ larvae at 120 hAEL were measured using ELISA. Average values of five independent data sets are shown with standard errors. Statistical significance was calculated using Student’s *t*-test (* *P* < 0.05). **(L–N)** 20E feeding rescues developmental arrest in TOR^DN^, *TOR* RNAi-1 and *TOR* RNAi-2 animals. TOR^DN^ (L), *TOR* RNAi-1 (M), and *TOR* RNAi-2 (N) animals were reared on 20E-containing (0.5 mg/g 20E) or control medium from 72 hAEL. Percentages of pupariated animals are shown at indicated stages. Numbers of animals tested are in parentheses. **(O and P)** The CW checkpoint in control-1. Percentages of pupariated control-1 and TOR^DN^ animals after starvation at a given time point (O) and weight (P) are shown. Mean percentages of three independent groups (10 larvae in each group) are shown with standard errors. **(Q)** Knockdown of *TOR* in the PG causes decrease in DNA content. The PGs (upper panels, outlined) and nuclei of PG cells (lower panels, outlined) were labeled for Dib (green) and DNA (white) at 120 hAEL. Scale bars, 50 μm (upper panels) and 10 μm (lower panels). **(R and S)** The C value (R) and number (S) of PG cells at 120 hAEL. Average C value in control-2 is normalized to 64C. Error bars represent standard errors. Numbers of animals tested are in parentheses. **(T)** PG cells expressing *CycE-1* transgene undergo endocycle. The PGs (upper panels, outlined) and nuclei of PG cells (lower panels, outlined) in control (*phm22 > +*) and CycE-1 (*phm22 > CycE-1*) larvae were labeled for Dib (green) and DNA (white) at 120 hAEL. Scale bars, 50 μm (upper panels) and 10 μm (lower panels). **(U and V)** The C value (U) and number (V) of PG cells at 120 hAEL. Average C value in control is normalized to 64C. Error bars represent standard errors. Numbers of animals tested are in parentheses. **(W)** Expression of *CycE* in the PG does not alter developmental timing. Percentages of pupariated animals are shown at indicated stages. Numbers of animals tested are in parentheses.
(TIF)

S5 Fig The CW checkpoint in continuous 18°C experiment. The CW checkpoint in control +Gal80^ts^ (*tub-Gal80*^*ts*^, *phm22 > +*) and TOR^DN^ +Gal80^ts^ (*tub-Gal80*^*ts*^, *phm22 > TOR*.*TED*) animals. Percentages of pupariated animals after starvation at a given time point (A) and weight (B) in the continuous 18°C experiment are shown. Mean percentages of three independent groups (10 larvae in each group) are shown with standard errors.
(TIF)

S6 Fig Detailed analyses of insulin and amino acid signaling pathways in the PG. **(A and B)** 20E feeding rescues developmental delay in RagA^DN^ and InR^DN^ animals. RagA^DN^ (A) and InR^DN^ (B) larvae were reared on 20E-containing (0.5 mg/g 20E) or control medium from 72 hAEL. Percentages of pupariated animals are shown at indicated stages. Numbers of animals tested are in parentheses. **(C and D)** The CW checkpoint in control +Gal80^ts^ and InR^CA^ +Gal80^ts^ animals. Percentages of pupariated animals after starvation at a given time point (C) and weight (D) in the continuous 18°C experiment are shown. Mean percentages of three independent groups (10 larvae in each group) are shown with standard errors. **(E)** The PGs (upper panels, outlined) and nuclei of PG cells (lower panels, outlined) under nutrient-rich condition at 18°C. The PGs were labeled for Dib (green) and DNA (white) at 192 hAEL. Scale bars, 50 μm (upper panels) and 10 μm (lower panels). **(F)** The C value of PG cells at 120 hAEL. Average C value in control +Gal80^ts^ is normalized to 64C. Error bars represent standard errors. Numbers of animals tested are in parentheses. **(G)** Developmental profile of animals fed on standard *Drosophila* medium continuously at 18°C. Percentages of pupariated animals are shown at indicated stages. Numbers of animals tested are in parentheses.
(TIF)

S1 Table Fly stocks used in this study. *BDSC, Bloomington *Drosophila* Stock Center; VDRC, Vienna *Drosophila* Resource Center.
(PDF)

S2 Table Fly stocks for RNAi experiment in this study. *BDSC, Bloomington Drosophila Stock Center; VDRC, Vienna Drosophila Resource Center.
(PDF)

S3 Table Genotypes of the flies used in this study. * Flies with *w*^*1118*^ were backcrossed with *w*^*1118*^ (BDSC #5905) three times. ** see S1 Table.
(PDF)

S4 Table The primer sets used for qPCR. (PDF)

## References

[1] Gordon CM, Laufer MR (2004) Physiology of puberty in *Pediatric and Adolescent Gynecology*, 5th Edition (eds. Emans S.J.H., Laufer M.R. and Goldstein D.P.), pp. 120–155. Philadelphia: Lippincott Williams & Wilkins.

[2] Frisch RE, Revelle R (1970) Height and weight at menarche and a hypothesis of critical body weights and adolescent events. Science 169: 397–399. 5450378 10.1126/science.169.3943.397

[3] Frisch RE, Revelle R (1971) Height and weight at menarche and a hypothesis of menarche. Arch Dis Child 46: 695–701. 5118059 10.1136/adc.46.249.695 PMC1647814

[4] Frisch RE (1987) Body fat, menarche, fitness and fertility. Hum Reprod 2: 521–533. 3117838 10.1093/oxfordjournals.humrep.a136582

[5] Callier V, Nijhout HF (2013) Body size determination in insects: a review and synthesis of size- and brain-dependent and independent mechanisms. Biol Rev Camb Philos Soc 88: 944–954. 10.1111/brv.12033 23521745

[6] Mirth CK, Riddiford LM (2007) Size assessment and growth control: how adult size is determined in insects. Bioessays 29: 344–355. 10.1002/bies.20552 17373657

[7] Rewitz KF, Yamanaka N, O'Connor MB (2013) Developmental checkpoints and feedback circuits time insect maturation. Curr Top Dev Biol 103: 1–33. 10.1016/B978-0-12-385979-2.00001-0 23347514 PMC4060521

[8] Yamanaka N, Rewitz KF, O'Connor MB (2013) Ecdysone control of developmental transitions: lessons from *Drosophila* research. Annu Rev Entomol 58: 497–516. 10.1146/annurev-ento-120811-153608 23072462 PMC4060523

[9] Mirth CK, Truman JW, Riddiford LM (2009) The ecdysone receptor controls the post-critical weight switch to nutrition-independent differentiation in *Drosophila* wing imaginal discs. Development 136: 2345–2353. 10.1242/dev.032672 19515698 PMC2729347

[10] Caldwell PE, Walkiewicz M, Stern M (2005) Ras activity in the *Drosophila* prothoracic gland regulates body size and developmental rate via ecdysone release. Curr Biol 15: 1785–1795. 10.1016/j.cub.2005.09.011 16182526

[11] Colombani J, Bianchini L, Layalle S, Pondeville E, Dauphin-Villemant C, Antoniewski C, et al (2005) Antagonistic actions of ecdysone and insulins determine final size in *Drosophila*. Science 310: 667–670. 10.1126/science.1119432 16179433

[12] Mirth C, Truman JW, Riddiford LM (2005) The role of the prothoracic gland in determining critical weight for metamorphosis in *Drosophila melanogaster*. Curr Biol 15: 1796–1807. 10.1016/j.cub.2005.09.017 16182527

[13] Layalle S, Arquier N, Léopold P (2008) The TOR pathway couples nutrition and developmental timing in *Drosophila*. Dev Cell 15: 568–577. 10.1016/j.devcel.2008.08.003 18854141

[14] Danielsen ET, Moeller ME, Yamanaka N, Ou Q, Laursen JM, Soenderholm C, et al (2016) A *Drosophila* Genome-Wide Screen Identifies Regulators of Steroid Hormone Production and Developmental Timing. Dev Cell 37: 558–570 10.1016/j.devcel.2016.05.015 27326933 PMC4918455

[15] Niwa R, Niwa YS (2014) Enzymes for ecdysteroid biosynthesis: their biological functions in insects and beyond. Biosci Biotechnol Biochem 78: 1283–1292. 10.1080/09168451.2014.942250 25130728

[16] Koyama T, Rodrigues MA, Athanasiadis A, Shingleton AW, Mirth CK (2014). Nutritional control of body size through FoxO-Ultraspiracle mediated ecdysone biosynthesis. Elife 3: e03091. 10.7554/eLife.03091 PMC4337420 25421296

[17] Niwa YS, Niwa R (2016) Transcriptional regulation of insect steroid hormone biosynthesis and its role in controlling timing of molting and metamorphosis. Dev Growth Differ 58: 94–105. 10.1111/dgd.12248 26667894 PMC11520982

[18] Edgar BA, Zielke N, Gutierrez C (2014) Endocycles: a recurrent evolutionary innovation for post-mitotic cell growth. Nat Rev Mol Cell Biol 15: 197–210. 10.1038/nrm3756 24556841

[19] Fox DT, Duronio RJ (2013) Endoreplication and polyploidy: insights into development and disease. Development 140: 3–12. 10.1242/dev.080531 23222436 PMC3513989

[20] Aggarwal SK, King RC (1969) Comparative study of the ring glands from wild type and *1(2)gl* mutant *Drosophila melanogaster*. J Morphol 129: 171–199. 10.1002/jmor.1051290204 5356774

[21] Welch RM (1957) A developmental analysis of the lethal mutant *l(2)gl* of *Drosophila melanogaster* based on cytophotometric determination of nuclear desoxyribonucleic acid (DNA) content. Genetics 42: 544–559. 17247715 10.1093/genetics/42.5.544 PMC1209849

[22] Havens CG, Walter JC (2011) Mechanism of CRL4 (Cdt2), a PCNA-dependent E3 ubiquitin ligase. Genes Dev 25: 1568–1582. 10.1101/gad.2068611 21828267 PMC3182024

[23] Shibutani ST, de la Cruz AF, Tran V, Turbyfill WJ 3rd, Reis T, Edgar BA, et al (2008) Intrinsic negative cell cycle regulation provided by PIP box- and Cul4Cdt2-mediated destruction of E2f1 during S phase. Dev Cell 15: 890–900. 10.1016/j.devcel.2008.10.003 19081076 PMC2644461

[24] Rewitz KF, Yamanaka N, Gilbert LI, O'Connor MB (2009) The insect neuropeptide PTTH activates receptor tyrosine kinase torso to initiate metamorphosis. Science 326: 1403–1405. 10.1126/science.1176450 19965758

[25] Sigrist SJ, Lehner CF (1997) *Drosophila* fizzy-related down-regulates mitotic cyclins and is required for cell proliferation arrest and entry into endocycles. Cell 90: 671–681. 9288747 10.1016/s0092-8674(00)80528-0

[26] Zhang H, Stallock JP, Ng JC, Reinhard C, Neufeld TP (2000) Regulation of cellular growth by the *Drosophila* target of rapamycin dTOR. Genes Dev 14: 2712–2724. 11069888 10.1101/gad.835000 PMC317034

[27] Hennig KM, Neufeld TP (2002) Inhibition of cellular growth and proliferation by dTOR overexpression in *Drosophila*. Genesis 34: 107–110. 10.1002/gene.10139 12324961

[28] Weiss A, Herzig A, Jacobs H, Lehner CF (1998) Continuous Cyclin E expression inhibits progression through endoreduplication cycles in *Drosophila*. Curr Biol. 8: 239–242. 9501988 10.1016/s0960-9822(98)70090-9

[29] McGuire SE, Le PT, Osborn AJ, Matsumoto K, Davis RL (2003) Spatiotemporal rescue of memory dysfunction in *Drosophila*. Science 302: 1765–1768. 10.1126/science.1089035 14657498

[30] Kim E, Goraksha-Hicks P, Li L, Neufeld TP, Guan KL (2008) Regulation of TORC1 by Rag GTPases in nutrient response. Nat Cell Biol 10: 935–945. 10.1038/ncb1753 18604198 PMC2711503

[31] Dong J, Pan D (2004) Tsc2 is not a critical target of Akt during normal *Drosophila* development. Genes Dev 18: 2479–2484. 10.1101/gad.1240504 15466161 PMC529535

[32] Pallares-Cartes C, Cakan-Akdogan G, Teleman AA (2012) Tissue-specific coupling between insulin/IGF and TORC1 signaling via PRAS40 in *Drosophila*. Dev Cell 22: 172–82. 10.1016/j.devcel.2011.10.029 22264732

[33] Radimerski T, Montagne J, Rintelen F, Stocker H, van der Kaay J, Downes CP, et al (2002) dS6K-regulated cell growth is dPKB/dPI(3)K-independent, but requires dPDK1. Nat Cell Biol 4: 251–255. 10.1038/ncb763 11862217

[34] Laplante M, Sabatini DM (2012) mTOR signaling in growth control and disease. Cell 149: 274–293. 10.1016/j.cell.2012.03.017 22500797 PMC3331679

[35] Russell RC, Fang C, Guan KL (2011) An emerging role for TOR signaling in mammalian tissue and stem cell physiology. Development 138: 3343–3356. 10.1242/dev.058230 21791526 PMC3143559

[36] Suzuki Y, Koyama T, Hiruma K, Riddiford LM, Truman JW (2013) A molt timer is involved in the metamorphic molt in *Manduca sexta* larvae. Proc Natl Acad Sci U S A 110: 12518–12525. 10.1073/pnas.1311405110 23852731 PMC3732944

[37] Yarosh W, Spradling AC (2014) Incomplete replication generates somatic DNA alterations within *Drosophila* polytene salivary gland cells. Genes Dev 28: 1840–1855. 10.1101/gad.245811.114 25128500 PMC4197960

[38] Nordman J, Li S, Eng T, Macalpine D, Orr-Weaver TL (2011) Developmental control of the DNA replication and transcription programs. Genome Res 21: 175–181. 10.1101/gr.114611.110 21177957 PMC3032921

[39] Demontis F, Perrimon N (2009) Integration of Insulin receptor/Foxo signaling and dMyc activity during muscle growth regulates body size in *Drosophila*. Development 136: 983–993. 10.1242/dev.027466 19211682 PMC2727562

[40] Ohhara Y, Shimada-Niwa Y, Niwa R, Kayashima Y, Hayashi Y, Akagi K, et al (2015) Autocrine regulation of ecdysone synthesis by β3-octopamine receptor in the prothoracic gland is essential for *Drosophila* metamorphosis. Proc Natl Acad Sci U S A 112: 1452–1457. 10.1073/pnas.1414966112 25605909 PMC4321272

[41] Yamanaka N, Marqués G, O'Connor MB (2015) Vesicle-mediated steroid hormone secretion in *Drosophila melanogaster*. Cell 163: 907–919. 10.1016/j.cell.2015.10.022 26544939 PMC4636736

[42] Hayashi Y, Hayashi M, Kobayashi S (2004) Nanos suppresses somatic cell fate in *Drosophila* germ line. Proc Natl Acad Sci U S A 101: 10338–10342. 10.1073/pnas.0401647101 15240884 PMC478573

[43] Rewitz KF, Yamanaka N, O'Connor MB (2010) Steroid hormone inactivation is required during the juvenile-adult transition in *Drosophila*. Dev Cell 19: 895–902. 10.1016/j.devcel.2010.10.021 21145504 PMC3025487

[44] Parvy JP, Blais C, Bernard F, Warren JT, Petryk A, Gilbert LI, et al (2005) A role for βFTZ-F1 in regulating ecdysteroid titers during post-embryonic development in *Drosophila melanogaster*. Dev Biol 282: 84–94. 10.1016/j.ydbio.2005.02.028 15936331

[45] Schindelin J, Arganda-Carreras I, Frise E, Kaynig V, Longair M, Pietzsch T, et al (2012) Fiji: an open-source platform for biological-image analysis. Nat Methods 9: 676–682. 10.1038/nmeth.2019 22743772 PMC3855844

