# Supplementary figures and images for: Nutrient-Dependent Endocycling in Steroidogenic Tissue Dictates Timing of Metamorphosis in Drosophila melanogaster
Source: PLoS Genet. 2017 Jan 25;13(1):e1006583. doi: 10.1371/journal.pgen.1006583 (PMC5298324; doi:10.1371/journal.pgen.1006583)

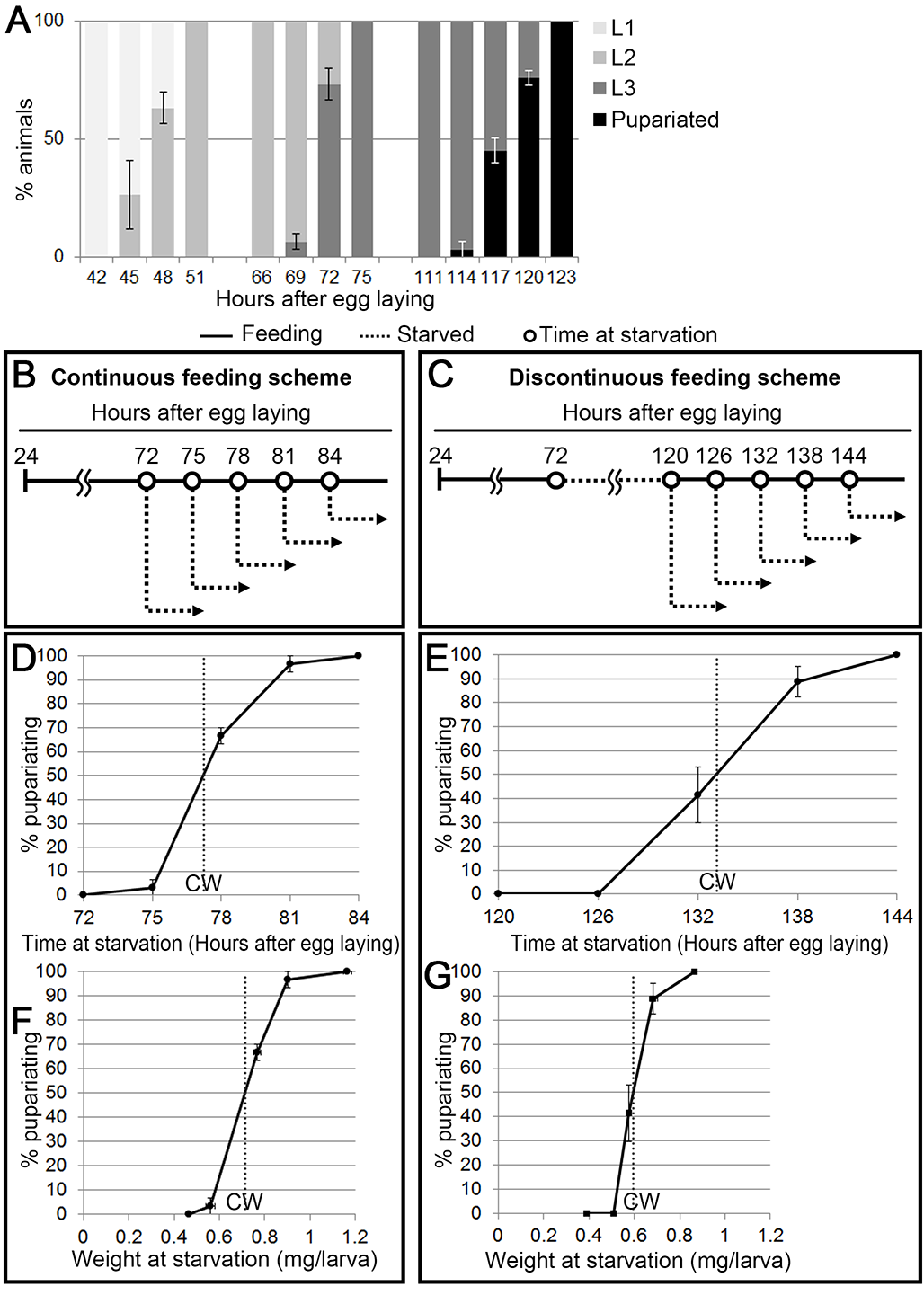

Supplement: S1 Fig — (A) Developmental profile of Oregon R flies. Percentages of larvae and pupariated animals are shown at indicated stages. Mean percentages of three independent groups (10 animals in each group) are shown with standard errors. (B and C) Schematic diagrams of the starvation experiments. In the continuous feeding scheme (B), larvae reared on standard Drosophila medium (black line) were starved on wet filter paper (dashed lines) from indicated time points (white circles). In the discontinuous feeding scheme (C), larvae starved on wet filter paper from 72 to 120 hAEL were transferred to standard Drosophila medium, and re-starved from indicated time points (white circle). For each time point, three independent groups (10 larvae in a group) were weighed before starvation, and pupariated animals were counted during starvation. (D–G) The CW checkpoint in Oregon R. Percentages of pupariated animals after starvation at a given time point (D and E) and weight (F and G) are shown in the continuous feeding (D and F) and discontinuous feeding scheme (E and G). Mean percentages of three independent groups (10 larvae in each group) are shown with standard errors. (TIF) [file pgen.1006583.s001.tif]

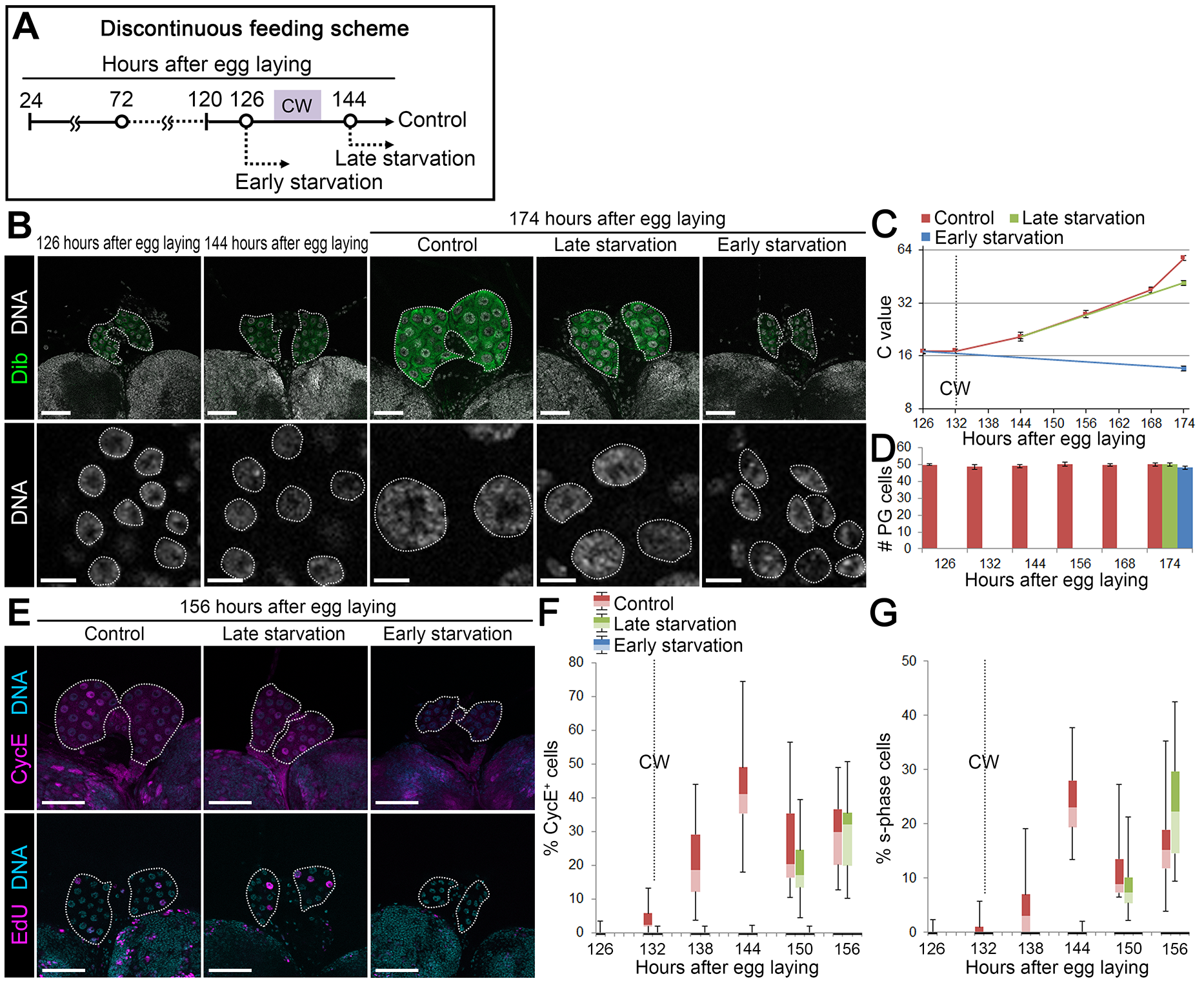

Supplement: S2 Fig — (A) Schematic diagram of the discontinuous feeding scheme using wild-type Oregon R flies. (B) Starvation before CW attainment causes arrest of DNA content increase in the PG. The PGs (upper panels, outlined) and nuclei of PG cells (lower panels, outlined) were labeled for Dib (green) and DNA (white) at indicated stages of the discontinuous feeding scheme. Scale bars, 50 μm (upper panels) and 10 μm (lower panels). (C and D) The C value (C) and number (D) of PG cells at indicated stages. The CW checkpoint in control is indicated by dashed line in C. The C value is normalized against that of the PG in control at 108 hAEL (see Fig 2B). Error bars represent standard errors. 10–17 PGs were analyzed for each group. (E) Starvation before CW attainment causes a decrease in CycE expression and EdU incorporation in the PG. The PGs were labeled for DNA (blue) and CycE or EdU (magenta) at 156 hAEL. The PGs are outlined by dashed lines. Scale bars, 50 μm. (F and G) Percentages of CycE-positive (F) and EdU-positive (G) s-phase PG cells at indicated stages. The CW checkpoint in control is indicated by dashed lines. 16–30 PGs were analyzed for each group. (TIF) [file pgen.1006583.s002.tif]

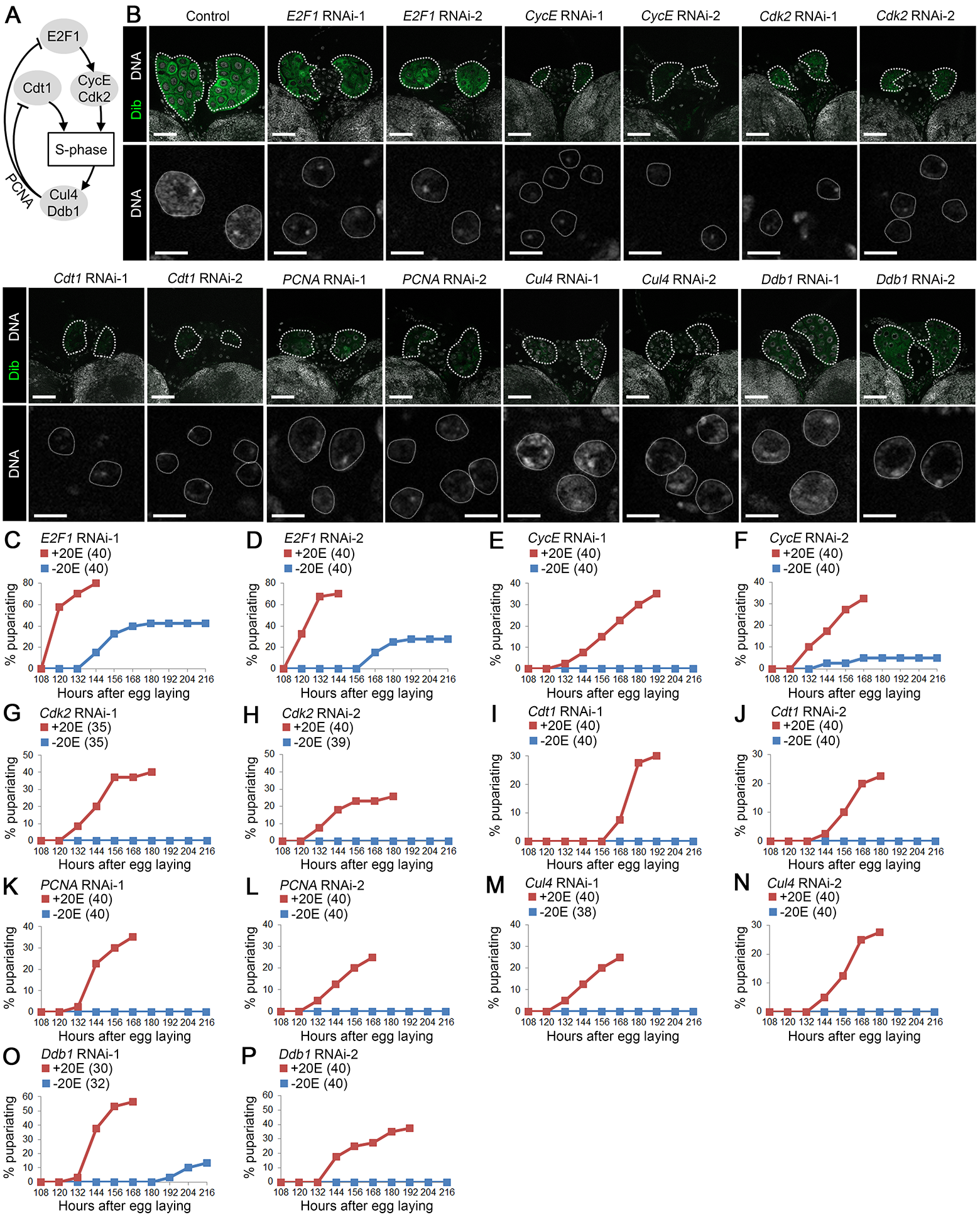

Supplement: S3 Fig — (A) Schematic diagram of interaction between endocycle regulators. (B) Knockdown of endocycle regulators in the PG causes reduction in DNA content. Each gene was knocked down using two independent RNAi lines. The PGs (upper panels, outlined) and nuclei of PG cells (lower panels, outlined) of control (phm22 > dicer2) and RNAi (phm22 > dicer2, RNAi) animals were labeled for Dib (green) and DNA (white) at 120 hAEL. Scale bars, 50 μm (upper panels) and 10 μm (lower panels). (C-P) E2F1 RNAi-1 (C) and 2 (D), CycE RNAi-1 (E) and 2 (F), Cdk2 RNAi-1 (G) and 2 (H), Cdt1 RNAi-1 (I) and 2 (J), PCNA RNAi-1 (K) and 2 (L), Cul4 RNAi-1 (M) and 2 (N), and Ddb1 RNAi-1 (O) and 2 (P) animals were reared on 20E-containing (0.5 mg/g 20E) or control medium from 72 hAEL. Percentages of pupariated animals are shown at indicated stages. Numbers of animals tested are in parentheses. (TIF) [file pgen.1006583.s003.tif]

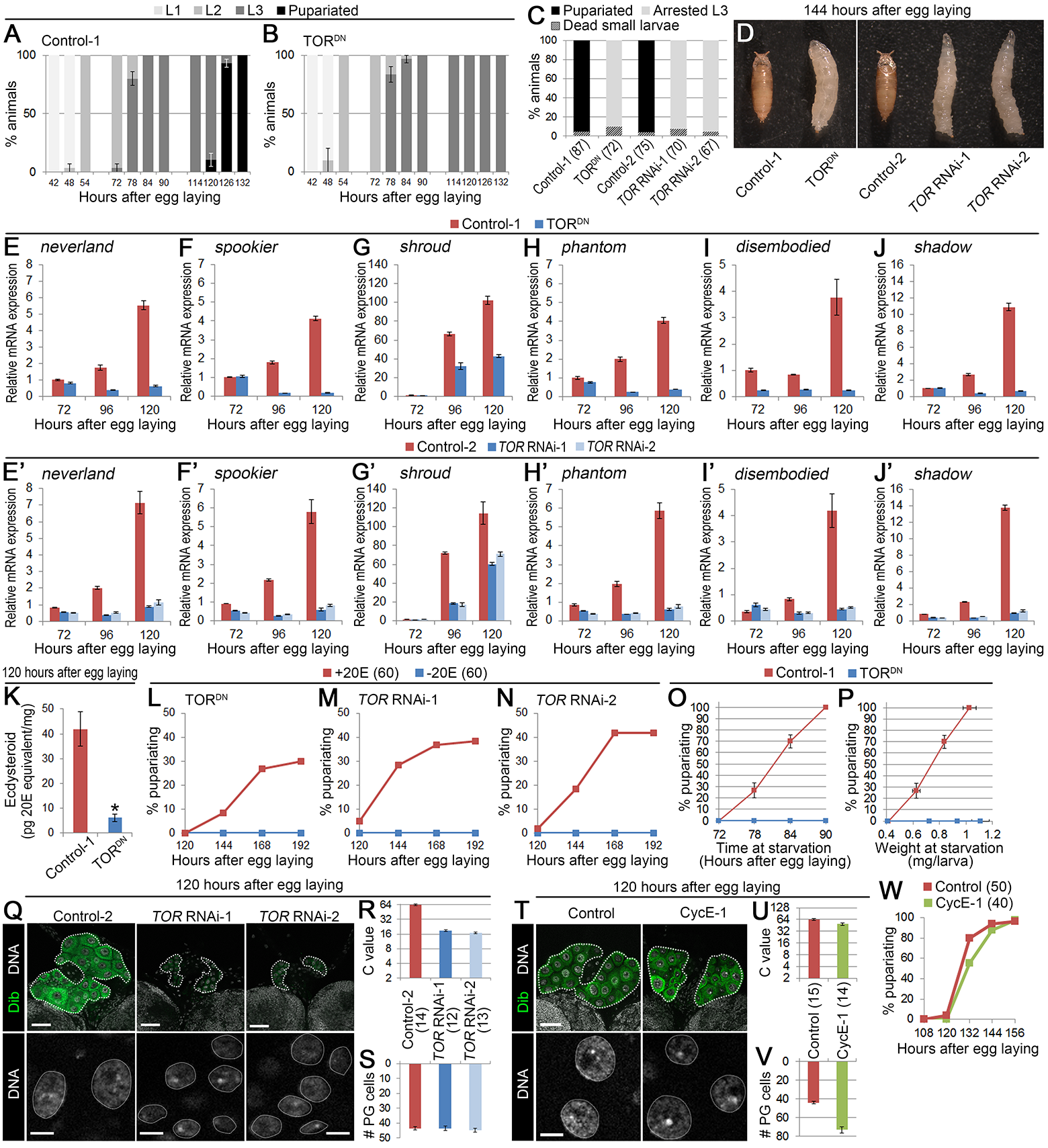

Supplement: S4 Fig — (A and B) Expression of TORDN in the PG causes developmental arrest at the 3rd instar larval stage. Percentages of larvae and pupariated animals in control-1 (phm22 > +) (A) and TORDN (phm22 > TOR.TED) (B) are shown at indicated stages. Mean percentages of three independent groups (10 larvae in each group) are shown with standard errors. (C) Knockdown of TOR in the PG causes developmental arrest at the 3rd instar larval stage. Developmental profiles of control-1, TORDN, control-2 (phm22 > dicer2), TOR RNAi-1 (phm22 > dicer2, TOR RNAi-1), and TOR RNAi-2 (phm22 > dicer2, TOR RNAi-2) animals are shown. Numbers of animals tested are in parentheses. (D) Pupariated control-1 and control-2 animals as compared to TORDN, TOR RNAi-1, and TOR RNAi-2 animals arrested at the 3rd instar larval stage. (E–J and E’–J’) Inhibition of TOR in the PG causes reduction in expression of ecdysone biosynthetic genes. Expression profiles of ecdysone biosynthetic genes were measured using qPCR. Average values of triplicate data sets are shown with standard errors. (K) Expression of TORDN in the PG causes decrease in ecdysteroid level. Whole-body ecdysteroid levels in control and TORDN larvae at 120 hAEL were measured using ELISA. Average values of five independent data sets are shown with standard errors. Statistical significance was calculated using Student’s t-test (* P < 0.05). (L–N) 20E feeding rescues developmental arrest in TORDN, TOR RNAi-1 and TOR RNAi-2 animals. TORDN (L), TOR RNAi-1 (M), and TOR RNAi-2 (N) animals were reared on 20E-containing (0.5 mg/g 20E) or control medium from 72 hAEL. Percentages of pupariated animals are shown at indicated stages. Numbers of animals tested are in parentheses. (O and P) The CW checkpoint in control-1. Percentages of pupariated control-1 and TORDN animals after starvation at a given time point (O) and weight (P) are shown. Mean percentages of three independent groups (10 larvae in each group) are shown with standard errors. (Q) Knockdown of TOR i [file pgen.1006583.s004.tif]

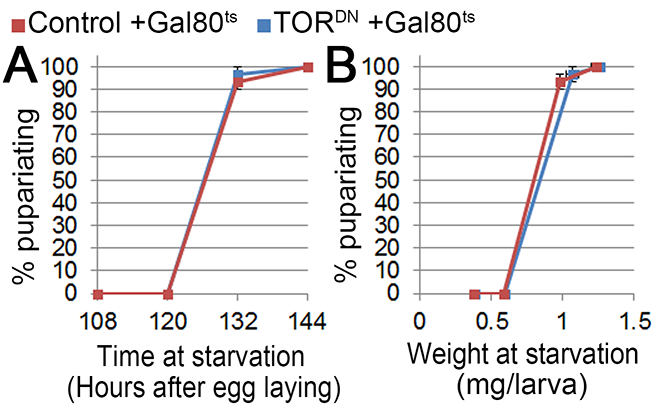

Supplement: S5 Fig — The CW checkpoint in control +Gal80ts (tub-Gal80ts, phm22 > +) and TORDN +Gal80ts (tub-Gal80ts, phm22 > TOR.TED) animals. Percentages of pupariated animals after starvation at a given time point (A) and weight (B) in the continuous 18°C experiment are shown. Mean percentages of three independent groups (10 larvae in each group) are shown with standard errors. (TIF) [file pgen.1006583.s005.tif]

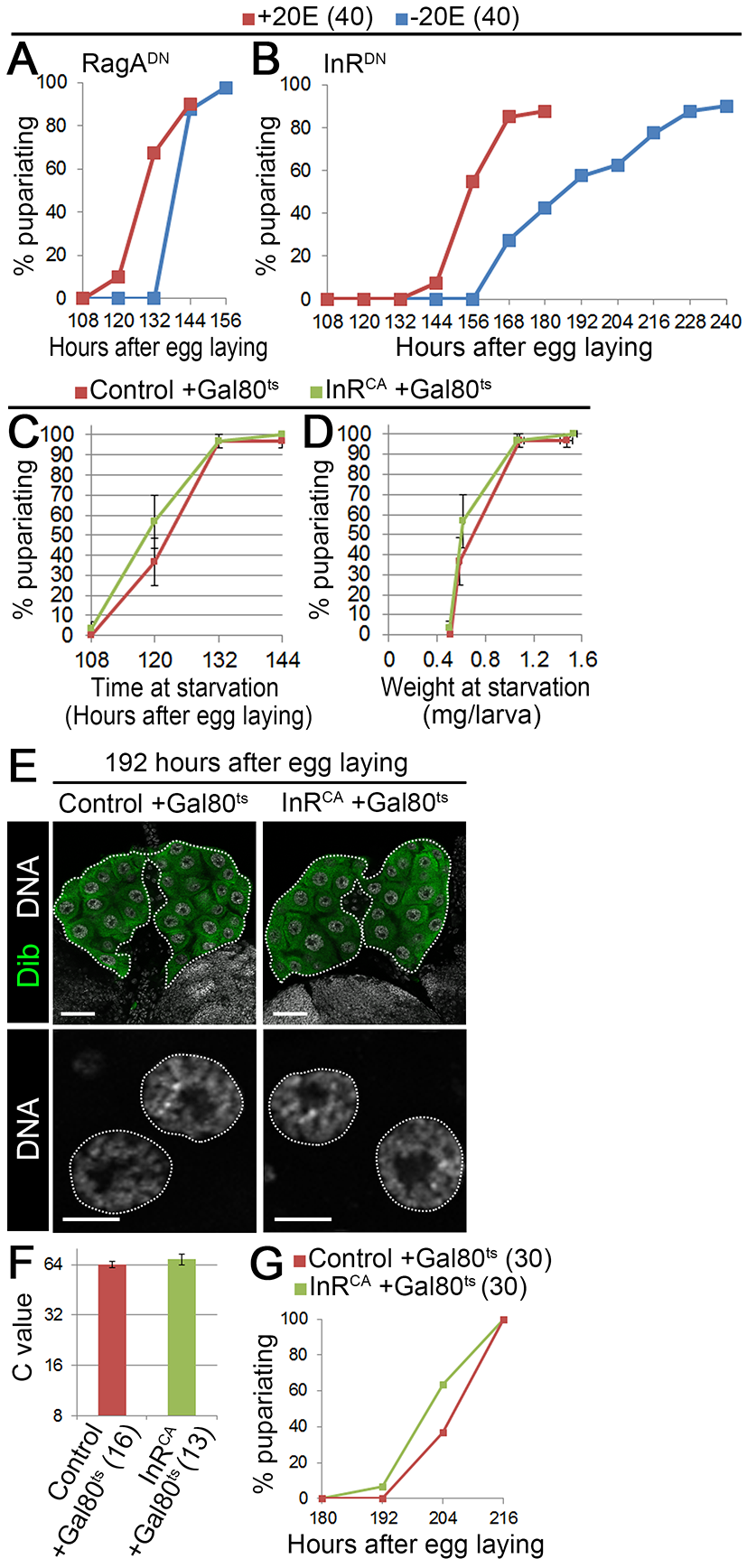

Supplement: S6 Fig — (A and B) 20E feeding rescues developmental delay in RagADN and InRDN animals. RagADN (A) and InRDN (B) larvae were reared on 20E-containing (0.5 mg/g 20E) or control medium from 72 hAEL. Percentages of pupariated animals are shown at indicated stages. Numbers of animals tested are in parentheses. (C and D) The CW checkpoint in control +Gal80ts and InRCA +Gal80ts animals. Percentages of pupariated animals after starvation at a given time point (C) and weight (D) in the continuous 18°C experiment are shown. Mean percentages of three independent groups (10 larvae in each group) are shown with standard errors. (E) The PGs (upper panels, outlined) and nuclei of PG cells (lower panels, outlined) under nutrient-rich condition at 18°C. The PGs were labeled for Dib (green) and DNA (white) at 192 hAEL. Scale bars, 50 μm (upper panels) and 10 μm (lower panels). (F) The C value of PG cells at 120 hAEL. Average C value in control +Gal80ts is normalized to 64C. Error bars represent standard errors. Numbers of animals tested are in parentheses. (G) Developmental profile of animals fed on standard Drosophila medium continuously at 18°C. Percentages of pupariated animals are shown at indicated stages. Numbers of animals tested are in parentheses. (TIF) [file pgen.1006583.s006.tif]
