# Supplementary material for: Nutrient-Dependent Endocycling in Steroidogenic Tissue Dictates Timing of Metamorphosis in Drosophila melanogaster
Source: PLoS Genet. 2017 Jan 25;13(1):e1006583. doi: 10.1371/journal.pgen.1006583 (PMC5298324; doi:10.1371/journal.pgen.1006583)
Supplement: S1 Table — *BDSC, Bloomington Drosophila Stock Center; VDRC, Vienna Drosophila Resource Center. (PDF) [file pgen.1006583.s007.pdf]

S1 Table. Fly stocks used in this study

Stock name	Source*	Stock#	Property
Oregon R	S. Kobayashi	-	
w^{1118}	BDSC	5905	
P{CaryP}attP2	BDSC	36303	
UAS-dicer2	VDRC	v60008	
UAS-CycE-1 (CycE.R)	BDSC	30725	Type1 CycE
UAS-TOR.TED	BDSC	7013	Dominant negative form of TOR
UAS-S6k.TE	BDSC	6912	Active form of S6k
UAS-RagA.T16N	T. P. Neufeld	-	Dominant negative form of RagA
UAS-InR.K1409A	BDSC	8253	Dominant negative form of InR
UAS-InR.A1325D	BDSC	8263	Active form of InR
UAS-mCD8::GFP	BDSC	5130	
$tub\text{-}Gal80^{ts}$	BDSC	7017	
$phantom^{22}\text{-}Gal4$	M. B. O'Connor	-	PG-selective Gal4 line
*BDSC, Bloomington *Drosophila* Stock Center; VDRC, Vienna *Drosophila* Resource Center			
