# Supplementary material for: Nutrient-Dependent Endocycling in Steroidogenic Tissue Dictates Timing of Metamorphosis in Drosophila melanogaster
Source: PLoS Genet. 2017 Jan 25;13(1):e1006583. doi: 10.1371/journal.pgen.1006583 (PMC5298324; doi:10.1371/journal.pgen.1006583)
Supplement: S2 Table — *BDSC, Bloomington Drosophila Stock Center; VDRC, Vienna Drosophila Resource Center. (PDF) [file pgen.1006583.s008.pdf]

S2 Table. Fly stocks for RNAi experiment in this study

Stock name	Target Gene	Source*	Stock#	Target site in gene region (bp)	Target length (bp)
UAS-E2F1 RNAi-1	*E2F1*	BDSC	27564	35137-35559	423
UAS-E2F1 RNAi-2	*E2F1*	VDRC	v15886	35176-35550	375
UAS-CycE RNAi-1	*CycE*	BDSC	29314	18149-18555	407
UAS-CycE RNAi-2	*CycE*	VDRC	v110204	18149-18487	339
UAS-Cdk2 RNAi-1	*Cdk2*	BDSC	36128	614-634	21
UAS-Cdk2 RNAi-2	*Cdk2*	VDRC	v104959	1367-1669	303
UAS-Cdt1 RNAi-1	*Cdt1*	BDSC	29562	2991-3458	468
UAS-Cdt1 RNAi-2	*Cdt1*	VDRC	v23131	3479-3788	310
UAS-PCNA RNAi-1	*PCNA*	VDRC	v51253	365-733	369
UAS-PCNA RNAi-2	*PCNA*	VDRC	v108384	467-854	388
UAS-Cul4 RNAi-1	*Cul4*	BDSC	50614	766-786	21
UAS-Cul4 RNAi-2	*Cul4*	VDRC	v105668	1268-1620	353
UAS-Ddb1 RNAi-1	*Ddb1*	BDSC	41997	2632-2652	21
UAS-Ddb1 RNAi-2	*Ddb1*	VDRC	v108924	2732-3397	666
UAS-Fzr RNAi	*Fzr*	VDRC	v25553	11002-11326	325
UAS-TOR RNAi-1	*TOR*	BDSC	34639	7341-7361	21
UAS-TOR RNAi-2	*TOR*	BDSC	33951	3633-3653	21
UAS-Rheb RNAi	*Rheb*	BDSC	33966	1464-1484	21
UAS-raptor RNAi	*raptor*	BDSC	34814	3566-3586	21
UAS-rictor RNAi-1	*rictor*	BDSC	31388	3266-3675	410
UAS-rictor RNAi-2	*rictor*	BDSC	31527	4890-5351	462
*BDSC, Bloomington *Drosophila* Stock Center; VDRC, Vienna *Drosophila* Resource Center					
