# Supplementary material for: Nutrient-Dependent Endocycling in Steroidogenic Tissue Dictates Timing of Metamorphosis in Drosophila melanogaster
Source: PLoS Genet. 2017 Jan 25;13(1):e1006583. doi: 10.1371/journal.pgen.1006583 (PMC5298324; doi:10.1371/journal.pgen.1006583)
Supplement: S3 Table — * Flies with w1118 were backcrossed with w1118 (BDSC #5905) three times. ** see S1 Table. (PDF) [file pgen.1006583.s009.pdf]

S3 Table. Genotypes of the flies used in this study

Name and properties	Abbreviated fly genotypes	Genotypes of parents*
Control (for RNAi experiment)	*phm22 > dicer2*	*w; UAS-dicer2; phantom*^{22}*-Gal4*<br>*y v;; attP2*
RNAi against genes of interest	*phm22 > dicer2, RNAi*	*w; UAS-dicer2; phantom*^{22}*-Gal4*<br>*UAS-RNAi***
Fzr RNAi +InR^{CA}	*phm22 > dicer2, Fzr RNAi, InR.A1325D*	*w; UAS-dicer2; phantom*^{22}*-Gal4*<br>*w; UAS-Fzr RNAi UAS-InR.A1325D*
Control	*phm22 > +*	*w*^{1118}*;; phantom*^{22}*-Gal4*<br>*w*^{1118}
TOR^{DN}	*phm22 > TOR.TED*	*w*^{1118}*;; phantom*^{22}*-Gal4*<br>*w*^{1118}*; UAS-TOR.TED*
Control +GFP	*phm22 > mCD8::GFP*	*w*^{1118}*;; phantom*^{22}*-Gal4 UAS-mCD8::GFP*<br>*w*^{1118}
TOR^{DN} +GFP	*phm22 > mCD8::GFP, TOR.TED*	*w*^{1118}*;; phantom*^{22}*-Gal4 UAS-mCD8::GFP*<br>*w*^{1118}*; UAS-TOR.TED*
TOR^{DN} +CycE-1	*phm22 > TOR.TED, CycE-1*	*w*^{1118}*;; phantom*^{22}*-Gal4*<br>*w*^{1118}*; UAS-TOR.TED UAS-CycE-1*
TOR^{DN} +S6k^{TE}	*phm22 > TOR.TED, S6k.TE*	*w*^{1118}*;; phantom*^{22}*-Gal4*<br>*w*^{1118}*; UAS-TOR.TED UAS-S6k.TE*
TOR^{DN} +CycE-1 +GFP	*phm22 > mCD8::GFP, TOR.TED, CycE-1*	*w*^{1118}*;; phantom*^{22}*-Gal4 UAS-mCD8::GFP*<br>*w*^{1118}*; UAS-TOR.TED UAS-CycE-1*
TOR^{DN} +S6k^{TE} +GFP	*phm22 > mCD8::GFP, TOR.TED, S6k.TE*	*w*^{1118}*;; phantom*^{22}*-Gal4 UAS-mCD8::GFP*<br>*w*^{1118}*; UAS-TOR.TED UAS-S6k.TE*
Control (for temperature-shift experiment)	*tub-Gal80*^{ts} *phm22 > +*	*w*^{1118}*;; phantom*^{22}*-Gal4 tub-Gal80*^{ts}<br>*w*^{1118}
TOR^{DN} +tub-Gal80^{ts}	*tub-Gal80*^{ts} *phm22 > TOR.TED*	*w*^{1118}*;; phantom*^{22}*-Gal4 tub-Gal80*^{ts}<br>*w*^{1118}*; UAS-TOR.TED*
RagA^{DN}	*phm22 > RagA.T16N*	*w*^{1118}*;; phantom*^{22}*-Gal4*<br>*w*^{1118}*; UAS-RagA.T16N*
InR^{DN}	*phm22 > InR.K1409A*	*w*^{1118}*;; phantom*^{22}*-Gal4*<br>*w*^{1118}*;; UAS-InR.K1409A*
InR^{CA}	*phm22 > InR.A1325D*	*w*^{1118}*;; phantom*^{22}*-Gal4*<br>*w*^{1118}*; UAS-InR.A1325D*
InR^{CA} +TOR^{DN}	*phm22 > InR.A1325D, TOR.TED*	*w*^{1118}*;; phantom*^{22}*-Gal4*<br>*w*^{1118}*; UAS-InR.A1325D UAS-TOR.TED*
InR^{CA} +Gal80^{ts}	*tub-Gal80*^{ts} *phm22 > InR.A1325D*	*w*^{1118}*;; phantom*^{22}*-Gal4 tub-Gal80*^{ts}<br>*w*^{1118}*; UAS-InR.A1325D*
Control (for epistasis analysis between *InR* and *Rheb*/*raptor*)	*phm22 > +*	*w*^{1118}*;; phantom*^{22}*-Gal4*<br>*y v;; attP2*
InR^{CA} (for epistasis analysis between *InR* and *Rheb*/*raptor*)	*phm22 > InR.A1325D*	*w*^{1118}*; UAS-InR.A1325D; phantom*^{22}*-Gal4/TM6B tub-Gal80*<br>*y v;; attP2*
InR^{CA} +RNAi (for epistasis analysis between *InR* and *Rheb*/*raptor*)	*phm22 > InR.A1325D, RNAi*	*w*^{1118}*; UAS-InR.A1325D; phantom*^{22}*-Gal4/TM6B tub-Gal80*<br>*UAS-RNAi***
* Flies with *w*^{1118} were backcrossed with *w*^{1118} (BDSC #5905) three times.		
** see S1 Table.		
