# Supplementary material for: Nutrient-Dependent Endocycling in Steroidogenic Tissue Dictates Timing of Metamorphosis in Drosophila melanogaster
Source: PLoS Genet. 2017 Jan 25;13(1):e1006583. doi: 10.1371/journal.pgen.1006583 (PMC5298324; doi:10.1371/journal.pgen.1006583)
Supplement: S4 Table — (PDF) [file pgen.1006583.s010.pdf]

S4 Table. The primer sets used for qPCR

gene name	forward primer sequence (5'-3')	reverse primer sequence (5'-3')
rp49	ACAAATGGCGCAAGCCCAAGG	ATGTGGCGGGTGCGCTTGTT
neverland	GGAAGCGTTGCTGACGACTGTG	TAAAGCCGTCCACTTCCTGCGA
spookier	TATCTCTTGGGCACACTCGCTG	GCCGAGCTAAATTTCTCCGCTT
shroud	CCACAACATCAAGTCGGAAGGAGC	ACCAGGCGAATGGAATCGGG
phantom	GGATTTCTTTCGGCGCGATGTG	TGCCTCAGTATCGAAAAGCCGT
disembodied	TGCCCTCAATCCCTATCTGGTC	ACAGGGTCTTCACACCCATCTC
shadow	CCGCATTCAGCAGTCAGTGG	ACCTGCCGTGTACAAGGAGAG
